# Heterocyclic 1,3-diazepine-based thio­nes and selones as versatile halogen-bond acceptors

**DOI:** 10.1107/S2052520622008150

**Published:** 2022-08-31

**Authors:** Arianna C. Ragusa, Andrew J. Peloquin, Marjan M. Shahani, Keri N. Dowling, James A. Golen, Colin D. McMillen, Daniel Rabinovich, William T. Pennington

**Affiliations:** a Department of Chemistry, Clemson University, 219 Hunter Laboratories, Clemson, SC 29634, USA; b Department of Chemistry, University of North Carolina at Charlotte, 9201 University City Blvd, Charlotte, NC 28223, USA; c Department of Chemistry and Biochemistry, University of Massachusetts Dartmouth, North Dartmouth, MA 02747, USA; d Joint School of Nanoscience and Nanoengineering, 2907 E. Gate City Blvd, Greensboro, NC 27401, USA; IISER Kolkata, India

**Keywords:** halogen bonding, thione, selone, organoiodine, cocrystal, diiodine

## Abstract

Utilizing the *N*-heterocyclic chalcogenones hexa­hydro-1,3-bis­(2,4,6-tri­methyl­phenyl)-2*H*-1,3-diazepine-2-thione (**SDiazMesS**) and hexa­hydro-1,3-bis­(2,4,6-tri­methyl­phenyl)-2*H*-1,3-diazepine-2-selone (**SDiazMesSe**) as halogen-bond acceptors, a total of 24 new cocrystals were prepared. The solid-state structures of the parent molecules were also determined, along with those of their aceto­nitrile solvates. Through the reaction of the chalcogen atom with molecular diiodine, a variety of S—I—I and Se—I—I fragments were formed, spanning a wide range of I—I bond orders. With acetone as a reaction solvent, molecular diiodine causes the oxidative addition of acetone to the chalcogen atom, resulting in new C—S, C—Se and C—C covalent bonds under mild conditions. The common halogen-bond donors, iodo­penta­fluoro­benzene, 1,2-, 1,3- and 1,4-di­iodo­tetra­fluoro­benzene, 1,3,5-tri­fluoro­tri­iodo­benzene and tetra­iodo­ethyl­ene resulted in halogen-bond-driven cocrystal formation. In most cases, the analogous **SDiazMesS** and **SDiazMesSe** cocrystals are isomorphic.

## Abstract 2

Utilizing the *N*-heterocyclic chalcogenones hexa­hydro-1,3-bis­(2,4,6-tri­methyl­phenyl)-2*H*-1,3-diazepine-2-thione (SDiazMesS) and hexa­hydro-1,3-bis­(2,4,6-tri­methyl­phenyl)-2*H*-1,3-diazepine-2-selone (SDiazMesSe) as halogen-bond acceptors, 24 new cocrystals were prepared. The solid-state structures of the parent molecules were also determined, along with those of their aceto­nitrile solvates.

## 1. Introduction

Halogen bonding has long been known, and formally defined by the International Union of Pure and Applied Chemistry (IUPAC) in 2013, as an attractive interaction between an electrophilic region on a halogen atom (halogen-bond donor) and a nucleophilic region on another atom or molecule (halogen-bond acceptor) (Desiraju *et al.*, 2013 ▸). The electrophilic region is referred to as the σ hole and is located at the ‘cap’ on the halogen end of the covalent bond, and is accompanied by a ‘belt’ of relatively higher electrostatic potential orthogonal to the bond (Murray *et al.*, 2009 ▸; Politzer *et al.*, 2010 ▸; Politzer & Murray, 2017 ▸). A similar electron density distribution is observed for the chalcogen atoms and is particularly pronounced for thio­nes and selones (Vogel *et al.*, 2019 ▸). If the chalcogen atoms of these functional groups act as halogen-bond acceptors, the location of the higher electrostatic potential drives the halogen bond away from the terminus of the thione or selone double bond. Conversely, a chalcogen bond, an interaction analogous to a halogen bond involving the σ hole of a chalcogen atom, can occur at the terminus of the thione or selone double bond (Aakeroy *et al.*, 2019 ▸).

As halogen-bond acceptor atoms, nitro­gen and oxygen have received considerably more attention than the heavier chalcogens. For example, a survey of the Cambridge Structural Database (CSD, Version 5.42, update 3; Groom *et al.*, 2016 ▸), limited to organics, yields 918 results involving an N⋯I halogen bond (where the N⋯I distance is less than the sum of the van der Waals radii of the two atoms) to a pyridine-based nitro­gen atom. A similar search with urea-, thio­urea- or seleno­urea-based acceptors yields 36, 100 and 19 results, respectively. Amongst the limited published data involving Se⋯I interactions, the oxidative addition of interhalogens to diselones, resulting in I—Se—X hypervalent systems is notable (Juárez-Pérez *et al.*, 2011 ▸). Our group has been particularly interested in the cooperation of halogen and chalcogen bonding as a versatile crystal engineering tool (Peloquin, McMillen *et al.*, 2021*a*, ▸ *b* ▸; Peloquin, McCollum *et al.*, 2021 ▸; Peloquin, Alapati *et al.*, 2021 ▸).

Motivated by the lack of published structural data involving halogen bonds to thio­ureas, and especially seleno­ureas, this work serves to further catalog the intermolecular interactions of these functionalities with common organoiodine compounds (Scheme 1 shows the organic halogen-bond acceptors and donors utilized in this study), as well as to investigate their reactivity and resulting halogen bonding with molecular diiodine. 
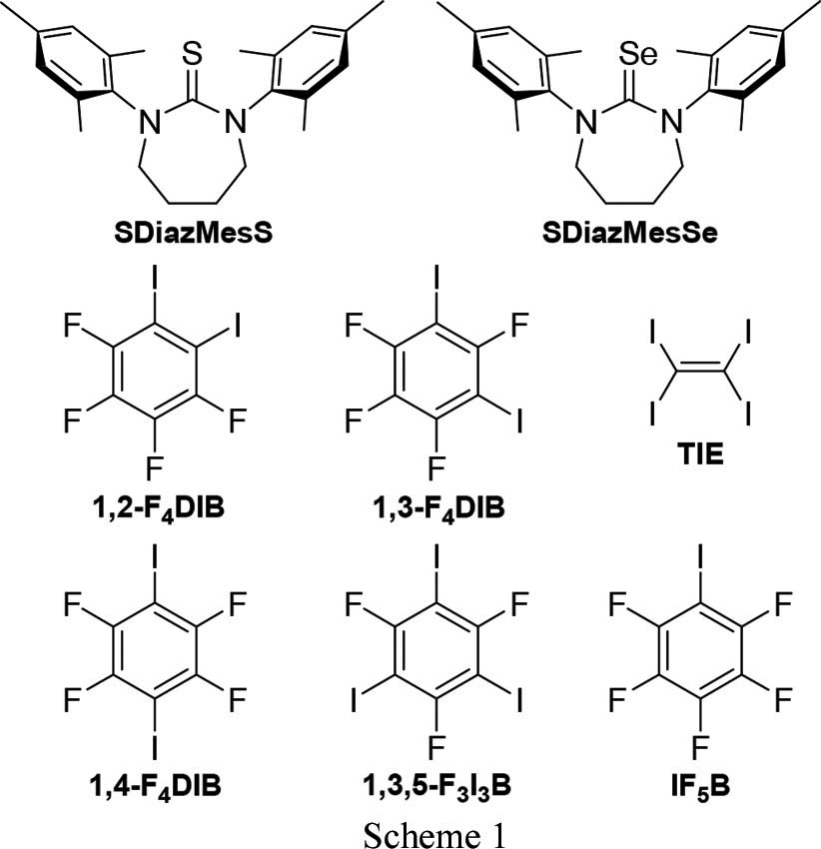


To this end, the sterically encumbered diazepine chalcogenone derivatives hexa­hydro-1,3-bis­(2,4,6-tri­methyl­phenyl)-2*H*-1,3-diazepine-2-thione (**SDiazMesS**) and hexa­hydro-1,3-bis­(2,4,6-tri­methyl­phenyl)-2*H*-1,3-diazepine-2-selone (**SDiazMesSe**) were prepared, the latter of which has not yet been reported in the synthetic literature, and structurally characterized. These were subsequently utilized as halogen-bond acceptors, to explore their halogen bonding tendencies. Each of these parent molecules was reacted with molecular diiodine, which depending on reaction stoichiometry and solvent choice, provided a variety of C=S—I—I, C=Se—I—I and C=Se—I derivatives. When acetone was utilized as the reaction solvent with I_2_, new S—C, Se—C and C—C bonds were formed via oxidation by I_2_. Utilizing the six most common commercially available halogen bond donors, 1,2-di­iodo­tetra­fluoro­benzene (1,2-F_4_DIB), 1,3-di­iodo­tetra­fluoro­benzene (1,3-F_4_DIB), 1,4-di­iodo­tetra­fluoro­benzene (1,4-F_4_DIB), 1,3,5-tri­fluoro­tri­iodo­benzene (1,3,5-F_3_I_3_B), iodo­penta­fluoro­benzene (IF_5_B), and tetra­iodo­ethyl­ene (TIE), the molecular structures of 14 new cocrystals were determined. In most cases, the analogous **SDiazMesS** and **SDiazMesSe** cocrystals are isomorphic. No significant chalcogen⋯chalcogen (ch⋯ch) or chalcogen⋯iodine (ch⋯I) chalogen bonding or I⋯F halogen bonding is observed within this series of structures.

## 2. Experimental

### 2.1. Materials and instrumentation

All reactions were performed under aerobic conditions unless otherwise stated. Solvents were purified and degassed by standard procedures, and all commercially available reagents were used as received. The bis­(mesityl) formamidine MesN = CHNHMes (MesForm) was synthesized as reported (Kuhn & Grubbs, 2008 ▸) and its corresponding diazepinium bromide derivative (SDiazMesH)Br (Kolychev *et al.*, 2009 ▸) was prepared by a modification of a literature procedure (Iglesias *et al.*, 2008 ▸). ^1^H and ^13^C NMR spectra were obtained on Jeol ECX-300 (300 MHz) or Jeol ECA-500 (500 MHz) FT spectrometers. Chemical shifts are reported in p.p.m. relative to SiMe_4_ (δ = 0 p.p.m.) and were referenced internally with respect to the solvent resonances (^1^H: δ 2.05 for *d*_5_-acetone; ^13^C: δ 29.84 for (CD_3_)_2_CO; coupling constants are given in hertz (Hz). IR spectra were recorded on a PerkinElmer Spectrum 100 spectrometer using an attenuated total reflectance (ATR) accessory and are reported in cm^−1^; relative intensities of the absorptions are indicated in parentheses (*v* = very strong, *s* = strong, *m* = medium, *w* = weak). Elemental analyses were determined by Atlantic Microlab, Inc. (Norcross, GA, USA).

For single-crystal X-ray analysis, crystals were mounted on low-background cryogenic loops using paratone oil. Data were collected at 100 K using Mo *K*α radiation (λ = 0.71073 Å) on a Bruker D8 Venture diffractometer with an Incoatec Iµs microfocus source and a Photon 2 detector. Diffraction data were collected using φ and ω scans and subsequently processed and scaled using the *APEX3* (*SAINT*/*SADABS*) (Bruker, 2017 ▸) software. The structures were solved with the *SHELXT* structure solution program and refined utilizing *SHELXL*, both incorporated in the *OLEX2* (v1.5) program package (Sheldrick, 2015*b* ▸,*a* ▸; Dolomanov *et al.*, 2009 ▸). Hydrogen atoms were placed in geometrically optimized positions using the appropriate riding models. In (**SDiazMesS**)·(MeCN), (**SDiazMesSe**)·(MeCN), (**SDiazMesS**)·(1,3-F_4_DIB), (**SDiazMesSe**)·(1,3-F_4_DIB), 2(**SDiazMesS**)·(1,3-F_4_DIB), (**SDiazMesS**)·(1,3,5-F_3_I_3_B), 2(**SDiazMesS**)·(TIE) and 2(**SDiazMesSe**)·(TIE), positional disorder of the C—C—C—C portion of the diazepine ring and/or a mesityl substituent was modeled in two parts, utilizing the SIMU restraint as appropriate.

### 2.2. Preparation of **SDiazMesS** and **SDiazMesSe**

The bis­(mesityl)formamidine MesN = CHNHMes (MesForm) was synthesized as reported (Kuhn & Grubbs, 2008 ▸) and its corresponding diazepinium bromide derivative (SDiazMesH)Br, **SDiazMesS** and **SDiazMesSe** were prepared by a modification of literature procedures (Iglesias *et al.*, 2008 ▸; Rais *et al.*, 2016 ▸).

#### 2.2.1. (SDiazMesH)Br

A stirred mixture of 1,4-di­bromo­butane (5.939 g, 27.506 mmol), the formamidine MesForm (7.000 g, 24.963 mmol), and potassium carbonate (1.989 g, 14.392 mmol) in aceto­nitrile (100 ml) was heated to reflux under argon for 24 h. The resulting solution was cooled to room temperature and concentrated under reduced pressure to ∼2 ml to give a very viscous tan-colored residue. Di­chloro­methane (25 ml) was added to the residue and the mixture stirred overnight, facilitating the separation of a fluffy, white precipitate. The resulting suspension was concentrated under reduced pressure to half volume, treated with cold di­ethyl ether (60 ml), and the sticky, peach-colored product was isolated by vacuum filtration and dried in vacuo for 24 h (7.820 g, 72%). ^1^H NMR data (in CDCl_3_): δ 2.27 (*s*, 6H, CH_3_), 2.41 (*s*, 12H, CH_3_), 2.56 (*m*, 4H, CH_2_), 4.65 (*m*, 4H, CH_2_), 6.94 (*s*, 4H, C_6_H_2_), 7.21 (*s*, 1H, NCHN); ^1^H NMR data (in *d*_6_-acetone): δ 2.28 (*s*, 6H, CH_3_), 2.45 (*s*, 12H, CH_3_), 2.55 (*m*, 4H, CH_2_), 4.52 (*m*, 4H, CH_2_), 7.05 (*m*, 4H, C_6_H_2_), 8.13 (*s*, 1H, NCHN).

#### 2.2.2. SDiazMesS

A stirred mixture of (SDiazMesH)Br (5.106 g, 12.292 mmol), elemental sulfur (0.433 g, 13.506 mmol) and potassium carbonate (2.208 g, 15.976 mmol) in *n*-propanol (75 ml) was heated to reflux for 48 h. The resulting yellow suspension was concentrated to ∼2 ml under reduced pressure to give a beige viscous residue. The product was extracted into di­chloro­methane (50 ml) and the extract was treated with activated carbon (∼1 g) and filtered. The pale orange filtrate was washed with deionized water (3 × 30 ml), and the organic phase was dried over magnesium sulfate (∼1 g) and filtered. Concentration of the solution under vacuum to ∼1 ml and addition of hexanes (20 ml) led to the precipitation of the pale brown product, which was separated by filtration and dried in vacuo for 24 h (3.543 g, 79%). Mp = 179–181 °C (dec.). NMR data (in *d*_6_-acetone): ^1^H δ 2.09 (*m*, 4H, CH_2_), 2.23 (*s*, 6H, CH_3_), 2.29 (*s*, 12H, CH_3_), 3.90 (*m*, 4H, CH_2_), 6.85 (*s*, 6H, C_6_H_2_); ^13^C δ 19.0 (*q*, ^1^ *J*_C–H_ = 126, 4C, CH_3_), 20.9 (*q*, ^1^ *J*_C–H_ = 128, 2C, CH_3_), 26.4 (*t*, ^1^ *J*_C–H_ = 127, 2C, CH_2_), 55.0 (*t*, ^1^ *J*_C–H_ = 138, 2C, NCH_2_), 130.1 (*d*, ^1^ *J*_C–H_ = 164, 4C, C_*m*_ in C_6_H_2_), 135.5 (*s*, 4C, C_*o*_ in C_6_H_2_), 136.5 (*s*, 2C, C_*p*_ in C_6_H_2_), 145.5 (*s*, 2C, C_*ipso*_ in C_6_H_2_), C=S not observed. IR data: 3141 (*w*), 2939 (*w*), 2916 (*m*), 2854 (*w*), 2726 (*w*), 1679 (*w*), 1643 (*s*), 1607 (*w*), 1551 (*w*), 1488 (*m*), 1478 (*m*), 1465 (*s*), 1426 (*m*), 1381 (*w*), 1369 (*m*), 1359 (*w*), 1330 (*w*), 1308 (*s*), 1287 (*vs*), 1267 (*m*), 1214 (*m*), 1200 (*w*), 1182 (*w*), 1148 (*w*), 1121 (*w*), 1102 (*w*), 1032 (*w*), 1011 (*w*), 997 (*w*), 979 (*w*), 957 (*w*), 914 (*w*), 863 (*w*), 849 (*s*), 767 (*m*), 748 (*w*), 740 (*w*), 729 (*w*), 709 (*w*). Anal. Calc.: for C_23_H_30_N_2_S: C, 75.4; H, 8.3; N, 7.6; found: C, 76.4; H, 8.2; N, 8.1%. Samples for single-crystal X-ray characterization were obtained from EtOH/DCM or MeCN.

#### 2.2.3. SDiazMesSe

A stirred mixture of (SDiazMesH)Br (7.386 g, 17.779 mmol), gray selenium (1.862 g, 23.582 mmol), and potassium carbonate (3.2190 g, 23.291 mmol) in *n*-propanol (150 ml) was heated to reflux for 20 h. The resulting dark red-orange suspension was concentrated to ∼2 ml under reduced pressure to give a dark orange solid residue. The product was extracted into di­chloro­methane (30 ml) and the bright yellow-orange extract was washed with DI water (3 × 30 ml). The organic phase was separated, dried over magnesium sulfate (∼1 g), filtered, concentrated under vacuum to ∼1 ml, and treated with cold hexanes (10 ml), leading to the precipitation of flaky, orange-yellow product, which was isolated by vacuum filtration and dried in vacuo for 14 h (4.457 g, 61%). Mp = 188–190 °C (dec.). NMR data (in *d*_6_-acetone): ^1^H δ 2.15 (*m*, 4H, CH_2_), 2.23 (*s*, 6H, CH_3_), 2.30 (*s*, 12H, CH_3_), 3.93 (*m*, 4H, CH_2_), 6.86 (*s*, 6H, C_6_H_2_); ^13^C δ 19.1 (*q*, ^1^ *J*_C–H_ = 127, 4C, CH_3_), 21.0 (*q*, ^1^ *J*_C–H_ = 127, 2C, CH_3_), 25.7 (*t*, ^1^ *J*_C–H_ = 128, 2C, CH_2_), 55.2 (*t*, ^1^ *J*_C–H_ = 139, 2C, NCH_2_), 130.2 (*d*, ^1^ *J*_C–H_ = 164, 4C, C_*m*_ in C_6_H_2_), 135.3 (*s*, 4C, C_*o*_ in C_6_H_2_), 136.7 (*s*, 2C, C_*p*_ in C_6_H_2_), 146.6 (*s*, 2C, C_*ipso*_ in C_6_H_2_), 186.7 (*s*, 1C, C=Se). IR data: 2971 (*w*), 2945 (*w*), 2916 (*w*), 2854 (*w*), 1737 (*m*), 1676 (*w*), 1645 (*s*), 1607 (*w*), 1550 (*w*), 1489 (*m*), 1471 (*s*), 1430 (*m*), 1369 (*m*), 1360 (*m*), 1331 (*m*), 1286 (*vs*), 1275 (*w*), 1254 (*w*), 1215 (*s*), 1203 (*w*), 1185 (*w*), 1149 (*w*), 1121 (*w*), 1104 (*w*), 1032 (*w*), 1012 (*w*), 998 (*w*), 981 (*w*), 902 (*w*), 864 (*w*), 850 (*s*), 775 (*w*), 755 (*w*), 743 (*w*), 707 (*w*). Anal. Calc.: for C_23_H_30_N_2_Se: C, 66.8; H, 7.3; N, 6.8; found: C, 66.7; H, 7.4; N, 6.7%. Samples for single-crystal X-ray characterization were obtained from EtOH/DCM or MeCN.

### 2.3. Reaction of **SDiazMesS** and **SDiazMesSe** with I_2_

#### 2.3.1. (**SDiazMesS**)I_2_

Di­ethyl ether (10 ml) was added to a mixture of **SDiazMesS** (0.150 g, 0.409 mmol) and elemental iodine (0.104 g, 0.405 mmol), resulting in the formation, within minutes, of a dark-orange solid and a dark-red solution. After stirring the suspension for 17 h, the product was isolated by filtration and dried in vacuo for 24 h (0.154 g, 61%). Mp = 141–143 °C (dec.). NMR data (in *d*_6_-acetone): ^1^H δ 2.27 (*s*, 10 H, CH_3_ + CH_2_), 2.37 (*s*, 12H, CH_3_), 4.12 (*s*, 4H, CH_2_) 6.96 (*s*, 4H, C_6_H_2_); ^13^C δ 18.5 (q, ^1^ *J*_C–H_ = 127, 4C, CH_3_), 20.5 (*q*, ^1^ *J*_C–H_ = 128, 2C, CH_3_), 23.7 (*t*, ^1^ *J*_C–H_ = 130, 2C, CH_2_), 54.7 (*t*, ^1^ *J*_C–H_ = 143, 2C, CH_2_), 129.6 (*d*, ^1^ *J*_C–H_ = 157, 4C, *C*_*m*_ in C_6_H_2_), 133.7 (*d*, ^2^ *J*_C–H_ = 6, 4C, C_*o*_ in C_6_H_2_), 136.7 (*s*, 2C, C_*p*_ in C_6_H_2_), 142.6 (*s*, 2C, C_*ipso*_ in C_6_H_2_), 176.3 (*s*, 1C, C=S). IR data: 2948 (*w*), 2910 (*w*), 2868 (*w*), 2730 (*w*), 1608 (*w*), 1506 (*s*), 1474 (*m*), 1452 (*w*), 1432 (*m*), 1391 (*m*), 1373 (*w*), 1365 (*w*), 1354 (*w*), 1337 (*m*), 1306 (*s*), 1286 (*vs*), 1270 (*vs*), 1211 (*w*), 1202 (*w*), 1187 (*w*), 1153 (*w*), 1105 (*w*), 1036 (*w*), 1014 (*w*), 999 (*w*), 978 (*w*), 966 (*w*), 937 (*w*), 926 (*w*), 910 (*w*), 892 (*w*), 853 (*s*), 841 (*m*), 798 (*w*), 756 (*m*), 745 (*w*), 729 (*w*), 706 (*w*). Anal. Calc.: for C_23_H_30_I_2_N_2_S: C, 44.5; H, 4.9; N, 4.5; found: C, 44.3; H, 4.9; N, 4.5%. Crystals suitable for X-ray diffraction analysis were obtained through the slow evaporation of an ethano­lic solution of the compound.

#### 2.3.2. (**SDiazMesSe**)I_2_

A mixture of **SDiazMesSe** (0.144 g, 0.349 mmol) and elemental iodine (0.093 g, 0.366 mmol) in di­ethyl ether (10 ml) was stirred overnight at room temperature. The resulting reddish-brown suspension was concentrated under reduced pressure to ∼1 ml, treated with di­ethyl ether (5 ml), and the dark orange product was isolated by filtration, washed with di­ethyl ether (2 ml), and dried in vacuo for 18 h (0.210 g, 91%). Mp = 195–198 °C (dec.). ^1^H NMR data (in *d*_6_-acetone): δ 2.28 (*s*, 3H, CH_3_), 2.33 (*s*, 4H, CH_2_), 2.37 (*s*, 6H, CH_3_), 4.22 (*s*, 4H, CH_2_), 6.98 (*s*, 2H, C_6_H_2_); ^13^C NMR data (in *d*_6_-DMSO): δ 17.9 (*q*, ^1^ *J*_C–H_ = 128, 4C, CH_3_), 20.0 (*q*, ^1^ *J*_C–H_ = 127, 2C, CH_2_), 22.6 (*t*, ^1^ *J*_C–H_ = 131, 2C, CH_2_), 55.5 (*t*, ^1^ *J*_C–H_ = 146, 2C, CH_2_), 129.5 (*d*, ^1^ *J*_C–H_ = 158, 2C, *C*_*m*_ in C_6_H_2_), 133.4 (*s*, ^1^ *J*_C–H_ = 160, 4C, *C*_*o*_ in C_6_H_2_), 138.1 (*s*, 4C, *C*_*p*_ in C_6_H_2_), 142.2 (*s*, 2C, *C*_*ipso*_ in C_6_H_3_), C=Se not observed. IR data: 2950 (*m*), 2911 (*m*), 2854 (*w*), 2730 (*w*), 1777 (*w*), 1739 (*w*), 1607 (*m*), 1516 (*s*), 1474 (*s*), 1452 (*w*), 1434 (*s*), 1392 (*m*), 1374 (*m*), 1365 (*m*), 1355 (*w*), 1338 (*m*), 1308 (*w*), 1293 (*vs*), 1282 (*vs*), 1269 (*vs*), 1261 (*vs*), 1210 (*m*), 1189 (*m*), 1150 (*w*), 1104 (*m*), 1035 (*m*), 998 (*m*), 979 (*w*), 957 (*w*), 938 (*w*), 923 (*w*), 896 (*w*), 853 (*s*), 739 (*m*), 704 (*w*). Anal. Calc.: for C_23_H_30_I_2_N_2_Se: C, 41.4; H, 4.5; N, 4.2; found: C, 41.1; H, 4.7; N, 4.1%. Crystals suitable for X-ray diffraction analysis were obtained through the slow evaporation of an ethano­lic solution of the compound.

### 2.4. Preparation of cocrystals

#### 2.4.1. (**SDiazMesS**)·(1,2-F_4_DIB)

In a 20 ml glass vial, **SDiazMesS** (30 mg, 0.082 mmol) and 1,2-F_4_DIB (33 mg, 0.082 mmol) were dissolved in a 1:1 mixture of ethanol and di­chloro­methane (5 ml) with gentle heating. The solvent was allowed to slowly evaporate under ambient conditions (18–20 °C) until colorless, needle-like crystals were observed.

#### 2.4.2. (**SDiazMesS**)·(1,3-F_4_DIB)

Using the same procedure as for (**SDiazMesS**)·(1,2-F_4_DIB), **SDiazMesS** (30 mg, 0.082 mmol) and 1,3-F_4_DIB (66 mg, 0.16 mmol) were combined to yield colorless, plate-like crystals.

#### 2.4.3. (**SDiazMesSe**)·(1,3-F_4_DIB)

Using the same procedure as for (**SDiazMesS**)·(1,2-F_4_DIB), **SDiazMesSe** (30 mg, 0.073 mmol) and 1,3-F_4_DIB (58 mg, 0.15 mmol) were combined to yield yellow, needle-like crystals.

#### 2.4.4. 2(**SDiazMesS**)·(1,3-F_4_DIB)

Using the same procedure as for (**SDiazMesS**)·(1,2-F_4_DIB), **SDiazMesS** (60 mg, 0.16 mmol) and 1,3-F_4_DIB (33 mg, 0.082 mmol) were combined to yield colorless, needle-like crystals.

#### 2.4.5. 2(**SDiazMesSe**)·(1,3-F_4_DIB)

Using the same procedure as for (**SDiazMesS**)·(1,2-F_4_DIB), **SDiazMesSe** (60 mg, 0.15 mmol) and 1,3-F_4_DIB (29 mg, 0.073 mmol) were combined to yield colorless, needle-like crystals.

#### 2.4.6. 2(**SDiazMesS**)·(1,4-F_4_DIB)^t^ and 2(**SDiazMesS**)·(1,4-F_4_DIB)^m^

Using the same procedure as (**SDiazMesS**)·(1,2-F_4_DIB), **SDiazMesS** (60 mg, 0.16 mmol) and 1,4-F_4_DIB (33 mg, 0.082 mmol) were combined to yield 2(**SDiazMesS**)·(1,4-F_4_DIB)^t^ as colorless, needle-like crystals on the sides of the vial and 2(**SDiazMesS**)·(1,4-F_4_DIB)^m^ as colorless, plank-like crystals from the bottom surface of the vial.

#### 2.4.7. 2(**SDiazMesSe**)·(1,4-F_4_DIB)^t^

Using the same procedure as (**SDiazMesS**)·(1,2-F_4_DIB), **SDiazMesSe** (60 mg, 0.15 mmol) and 1,4-F_4_DIB (29 mg, 0.073 mmol) were combined to yield colorless, plank-like crystals.

#### 2.4.8. (**SDiazMesS**)·(1,3,5-F_3_I_3_B)

Using the same procedure as (**SDiazMesS**)·(1,2-F_4_DIB), **SDiazMesS** (30 mg, 0.082 mmol) and 1,3,5-F_3_I_3_B (42 mg, 0.082 mmol) were combined to yield colorless, needle-like crystals.

#### 2.4.9. (**SDiazMesSe**)·(1,3,5-F_3_I_3_B)

Using the same procedure as (**SDiazMesS**)·(1,2-F_4_DIB), **SDiazMesSe** (30 mg, 0.073 mmol) and 1,3,5-F_3_I_3_B (37 mg, 0.073 mmol) were combined to yield yellow, needle-like crystals.

#### 2.4.10. (**SDiazMesS**)·(IF_5_B)

Using the same procedure as (**SDiazMesS**)·(1,2-F_4_DIB), **SDiazMesS** (30 mg, 0.082 mmol) and IF_5_B (120 mg, 0.41 mmol) were combined to yield colorless, plate-like crystals.

#### 2.4.11. 2(**SDiazMesSe**)·5(IF_5_B)

In a 1 ml glass tube, **SDiazMesSe** (30 mg, 0.073 mmol) was dissolved in IF_5_B (0.25 ml, 1.8 mmol). The solvent was allowed to slowly evaporate under ambient conditions, yielding yellow, plate-like crystals after approximately one week.

#### 2.4.12. 2(**SDiazMesS**)·(TIE)

Using the same procedure as for (**SDiazMesS**)·(1,2-F_4_DIB), **SDiazMesS** (60 mg, 0.16 mmol) and TIE (44 mg, 0.082 mmol) were combined to yield yellow, plate-like crystals.

#### 2.4.13. 2(**SDiazMesSe**)·(TIE)

Using the same procedure as for (**SDiazMesS**)·(1,2-F_4_DIB), **SDiazMesSe** (60 mg, 0.15 mmol) and TIE (39 mg, 0.073 mmol) were combined to yield yellow, block-like crystals.

## 3. Results and discussion

### 3.1. Synthesis of **SDiazMesS** and **SDiazMesSe**

The *N*-heterocyclic chalcogenones SDiazMes*E* (*E* = S, Se), envisioned to have good solubility in common organic solvents and exhibit simple ^1^H and ^13^C NMR spectra to facilitate characterization of products, were synthesized in three steps (Scheme 2). 
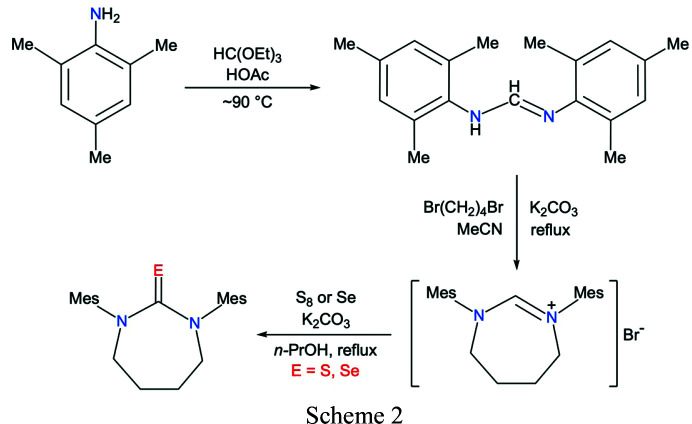
 .

Commercially available 2,4,6-tri­methyl­aniline (mesityl­amine) was reacted neat with tri­ethyl­orthoformate in the presence of a catalytic amount of acetic acid (Kuhn & Grubbs, 2008 ▸) to produce the bis­(mesityl)formamidine MesN=CHNHMes (MesForm) in ∼80% yield. The formamidine was then reacted with 1,4-di­bromo­butane and potassium carbonate in refluxing aceto­nitrile (Iglesias *et al.*, 2008 ▸) to generate the diazepinium bromide derivative (SDiazMesH)Br, which was isolated in ∼70% yield. Finally, the diazepinium salt is reacted with either elemental sulfur or gray selenium in the presence of a base (K_2_CO_3_) in refluxing *n*-propanol to form the desired thione and selone products in 60–80% yield. The two chalcogenones were fully characterized by a combination of analytical and spectroscopic techniques, including elemental analysis, IR, and ^1^H and ^13^C NMR spectroscopies, and single-crystal X-ray diffraction, as described in the next section.

### 3.2. Structure of **SDiazMesS** and **SDiazMesSe** and their MeCN solvates

**Figure 1:**
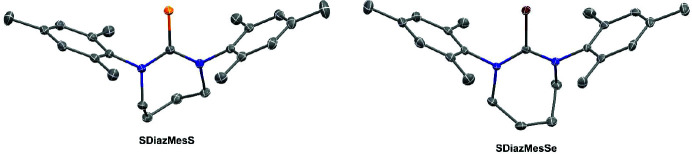
Views of the molecular structures of **SDiazMesS** (left-hand view) and **SDiazMesSe** (right-hand view). Displacement ellipsoids are drawn at the 50% probability level. Hydrogen atoms are omitted for clarity.

The parent compound **SDiazMesS** crystallizes in the monoclinic space group *P*2_1_/*c*, whereas **SDiazMesSe** crystallizes in the orthorhombic space group *Pbca* (Fig. 1 ▸). The thio­urea and seleno­urea are prominent in the respective structures, with a C=S length of 1.6887 (13) Å and an average C=Se length of 1.850 (4) Å from the two unique molecules in the asymmetric unit (Table 1 ▸). Despite the cyclic nature of the molecule’s core, the N—C=*E* (*E* = S, Se) angle remains at approximately 120°, consistent with that of an isolated thio­urea or seleno­urea molecule (Tomkowiak & Katrusiak, 2018 ▸). In **SDiazMesS**, weak C—H⋯S hydrogen bonds [C⋯S = 3.7030 (15) Å and 3.4858 (14) Å], involving hydrogen atoms of the heterocyclic ring, contribute to the stacking of molecules along the *c* axis. With two unique molecules in the asymmetric unit of **SDiazMesSe**, the hydrogen-bonding pattern is more complex. To one selenium atom, weak C—H⋯Se hydrogen bonds [C⋯Se = 3.671 (2) Å and 3.665 (2) Å] are observed to two different molecules, both involving hydrogen atoms of the heterocyclic rings. In both cases, the mesityl rings are nearly perpendicular to the urea plane. For example, in **SDiazMesS**, the mesityl-to-urea plane angles are 91.46 (4)° and 89.60 (4)°. These geometric parameters remain consistent throughout the variety of cocrystals, adducts, and solvates in this study. Unlike the unsolvated structures of the parent molecules, the aceto­nitrile solvates **(SDiazMesS)·(MeCN)** and **(SDiazMesSe)·(MeCN)** are isomorphous, with a weak hydrogen bonding interaction observed between the chalcogen atom and the aceto­nitrile molecule.

### 3.3. Reaction of **SDiazMesS** and **SDiazMesSe** with I_2_

The reaction of molecular iodine with **SDiazMesS** and **SDiazMesSe** provided a rich series of products depending on the ratio of I_2_ to thione or selone and the solvent choice (Scheme 3). 
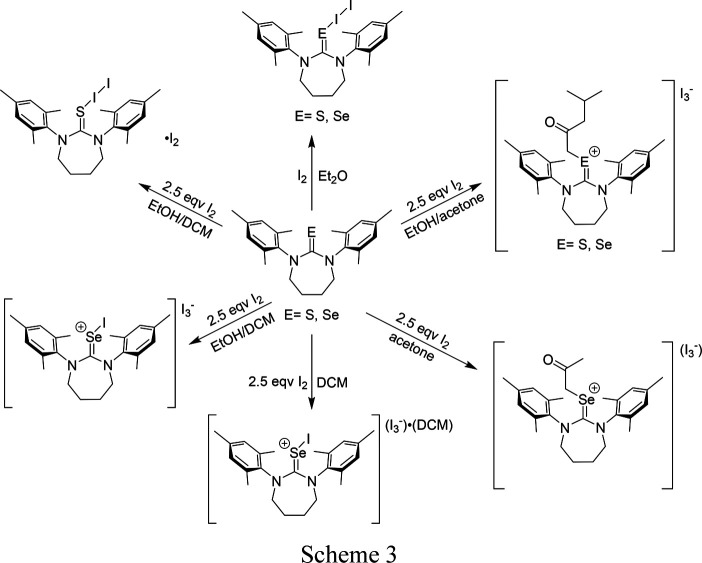


The reaction of **SDiazMesS** or **SDiazMesSe** with a stoichiometric amount of I_2_ in di­ethyl ether provides **SDiazMesS-I_2_** or **SDiazMesSe-I_2_**, both crystallizing in the triclinic space group 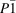 . In both cases, short chalcogen⋯iodine distances are observed [S—I = 2.6738 (9) Å and Se—I = 2.7559 (4) Å], with concomitant lengthening of the I—I bond to 2.8794 (4) Å in **SDiazMesS-I_2_** and 2.9106 (4) Å in **SDiazMesSe-I_2_**. These I—I distances correspond to bond orders of 0.59 and 0.53, respectively, as calculated using the expression of Pauling, *D*(*n*′) = *D*(1) − 0.71 log (*n*′) with a *D*(1) of 2.72 Å (Pauling, 1960 ▸). A series of weak C—H⋯I hydrogen bonds consolidate the packing.

**Figure 2:**
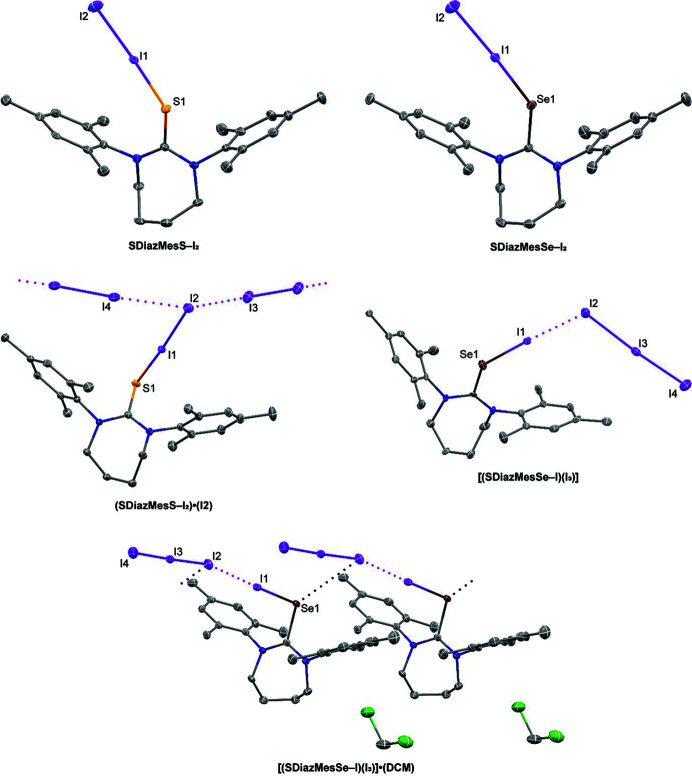
Views of the molecular structures of the products of the reaction of I_2_ with **SDiazMesS** and **SDiazMesSe** in non-acetone solvents. Intermolecular I⋯I and Se⋯I interactions are indicated by magenta and black dotted lines, respectively. Displacement ellipsoids are drawn at the 50% probability level. Hydrogen atoms are omitted for clarity.

The reaction with 2.5 molar equivalents of I_2_ in a 1:1 mixture of ethanol and di­chloro­methane provides different products from **SDiazMesS** and **SDiazMesSe**. When the reaction was conducted with **SDiazMesS**, the cocrystal **(SDiazMesS—I_2_)·(I_2_)** was obtained in the triclinic space group 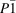 . The bond distances within the S—I—I fragment are reduced from those in **SDiazMesS**, with the S—I distance shrinking to 2.5052 (5) Å while the I–I distance further elongates to 3.0803 (4) Å. This change in bond geometries is indicative of further progression towards the dipolar I^+^⋯I^−^ extreme, with a calculated bond order of 0.31. The consolidation of the negative charge on the terminal iodine atom of the S—I—I fragment contributes to its increased ability to serve as a halogen-bond acceptor. The incorporation of a diiodine molecule within the structure aids in the formation of chains in the 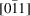 direction through I⋯I halogen bonding. There are two such unique I⋯I halogen bonds formed at each terminal iodine atom of the S—I—I, both of which are similar in length [I⋯I = 3.4171 (4) Å and 3.4694 (5) Å]. In contrast to the reaction of **SDiazMesS** with excess I_2_, the reaction of 2.5 molar equivalents of I_2_ with **SDiazMesSe** in 1:1 ethanol:dichloromethane provides the salt **[(SDiazMesSe—I)(I_3_)]**. Crystalline **[(SDiazMesSe—I)(I_3_)]** forms within five minutes, and if this material is recrystallized from di­chloro­methane, the crystalline solvate **[(SDiazMesSe—I)(I_3_)]·(DCM)** is obtained. In both cases, the Se—I—I fragment is better represented as (Se—I^+^)(I_3_^−^). This assignment is supported by the further contraction of the Se–I distance to 2.5807 (14) Å and expansion of the I^+^⋯I distance to 3.2052 (10) Å relative to **SDiazMesSe—I_2_**. This I⋯I distance would correspond to a calculated bond order of only 0.20. The C=Se length remains relatively unchanged [1.921 (10) Å] compared to **SDiazMesSe—I_2_** (1.8973 (16) Å. The triiodide anion in both salts is asymmetric, with the two I—I lengths of 3.0222 (10) Å and 2.8492 (10) Å in **[(SDiazMesSe—I)(I_3_)]** and 3.0098 (6) Å and 2.8426 (6) Å in **[(SDiazMesSe—I)(I_3_)]·(DCM)**. This degree of asymmetry is in line with other reported triiodide salts (Kobra *et al.*, 2018 ▸). In **[(SDiazMesSe—I)(I_3_)]**, halogen bonding does not contribute to the long-range packing motif, beyond the aforementioned connection of one end of I_3_^−^ to the Se—I fragment; however, in **[(SDiazMesSe—I)(I_3_)]·(DCM)**, a combination of I⋯I halogen bonding and Se⋯I chalcogen bonding contributes to the formation of chains along the *a* axis (Fig. 2 ▸).

**Figure 3:**
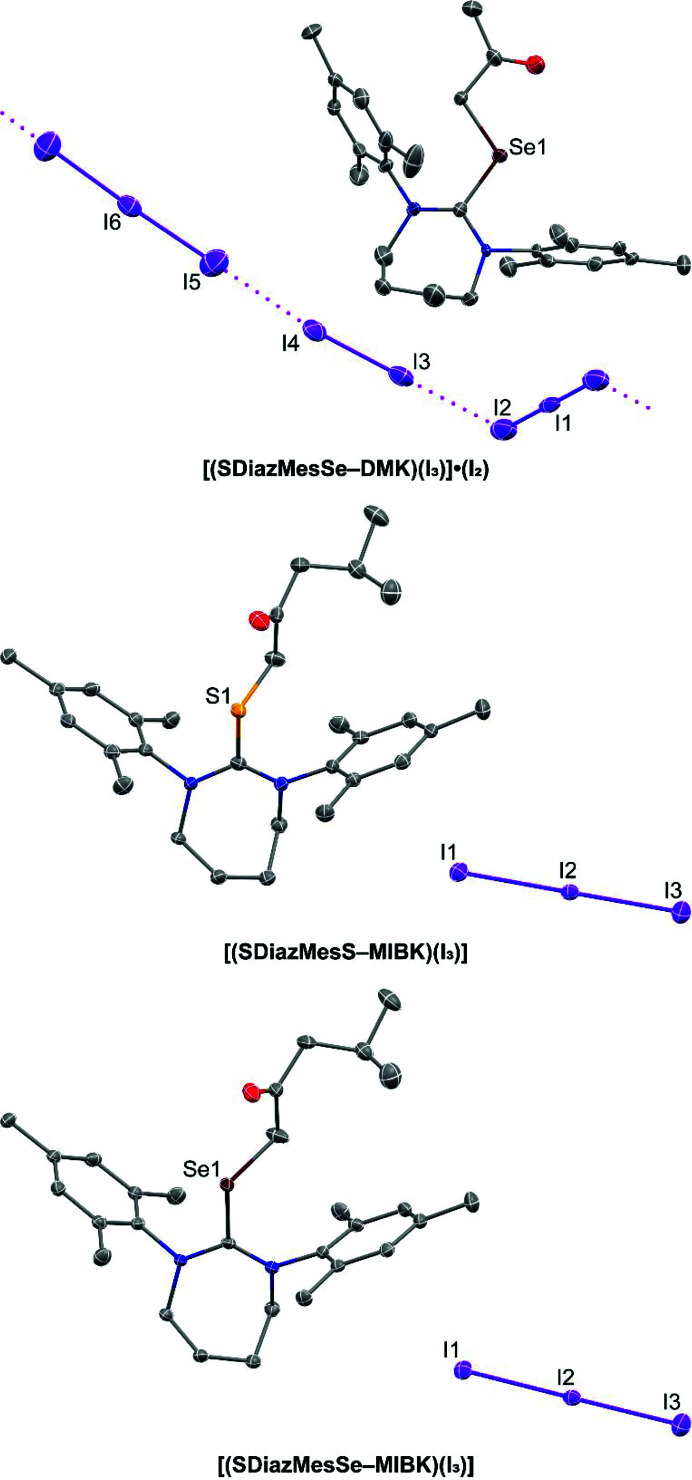
Views of the molecular structures of the products of the reaction of I_2_ with **SDiazMesS** and **SDiazMesSe** in acetone. Intermolecular I⋯I interactions are indicated by magenta dotted lines. Displacement ellipsoids are drawn at the 50% probability level. Hydrogen atoms are omitted for clarity.

The use of acetone as the reaction solvent allowed access to new organic products resulting from forming a new covalent bond between the chalcogen atom and a methyl carbon of acetone (Fig. 3 ▸). When the reaction of **SDiazMesSe** and 2.5 molar equivalents of diiodine was conducted in acetone, the salt **[(SDiazMesSe-DMK)(I_3_)]·(I_2_)** was obtained, displaying an added di­methyl­ketone (DMK) fragment, resulting from the diiodine-promoted addition of an acetone molecule to the selenium atom. The slight elongation of the C=Se bond and negligible change in the C—N lengths suggest the positive charge is primarily localized to the selenium atom (Table 1 ▸). If a 1:1 mixture of ethanol and acetone was utilized as the reaction solvent for the reaction with 2.5 molar equivalents of I_2_, the isomorphic products **[(SDiazMesS-MIBK)(I_3_)]** and **[(SDiazMesSe-MIBK)(I_3_)]** were obtained, both crystallizing in the triclinic space group 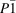 . The methyl­iso­butyl­ketone (MIBK) fragment bound to the chalcogen atom results from the further bond formation of the methyl carbon of **[(SDiazMesSe-DMK)(I_3_)]·(I_2_)** with the carbonyl carbon of an additional acetone molecule along with de­oxy­genation. A related reaction involving the addition of acetone to a sulfur atom in 1,4-di­thiane has been previously reported (Peloquin, Alapati *et al.*, 2021 ▸). Just as in **[(SDiazMesSe-DMK)(I_3_)]·(I_2_)**, the only slight lengthening of C—S and C—Se distances, along with a negligible change in C—N distances, relative to the parent molecule indicate the positive charge is primarily localized on the chalcogen atom. The triiodide anion is pinned in place by weak type I halogen bonds with the I_2_ molecule. While all attempts to isolate the analogous **SDiazMesS**-containing structure to **[(SDiazMesSe-DMK)(I_3_)]·(I_2_)** were unsuccessful, the isolation of **[(SDiazMesS-MIBK)(I_3_)]** does suggest its formation occurs. Adding ethanol to the reaction mixture reduces the overall solvent polarity and likely supports the solubility of the increased aliphatic character of the MIBK fragment over DMK.

### 3.4. Cocrystallization of **SDiazMesS** and **SDiazMesSe** with iodo­fluoro­benzenes

**Figure 4:**
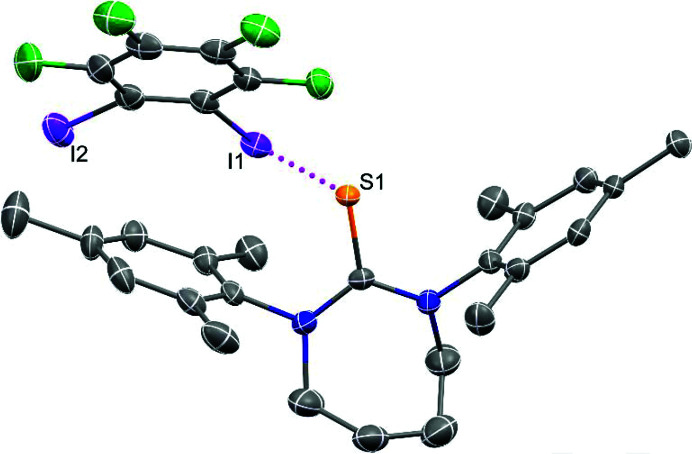
Halogen bonding in **(SDiazMesS)·(1,2-F_4_DIB)**. Intermolecular I⋯S halogen bonding is indicated by a magenta dotted line. Displacement ellipsoids are drawn at the 50% probability level. Hydrogen atoms are omitted for clarity.

The cocrystal **(SDiazMesS)·(1,2-F_4_DIB)** crystallizes in the orthorhombic space group *Pna*2_1_ with one molecule each of **SDiazMesS** and 1,2-F_4_DIB within the asymmetric unit (Fig. 4 ▸). C—I⋯S halogen bonding occurs between the thione sulfur atom and only one iodine atom of 1,2-F_4_DIB, leading to the formation of discrete halogen bonded dimers. The halogen-bond distance in this cocrystal, 3.2092 (12) Å, is significantly shorter than measured in the ternary cocrystal of thio­urea, 1,2-F4DIB, and 18-crown-6 [3.4680 (6) Å] (Topić & Rissanen, 2016 ▸). The lack of I⋯S halogen bonding to the second iodine atom is likely due to a combination of the steric bulk of **SDiazMesS** and the proximity of the iodine atoms in 1,2-F_4_DIB. Neighboring dimers consolidate through a combination of weak C—H⋯I and C—H⋯S interactions. All attempts to isolate the corresponding **SDiazMesSe** cocrystal were unsuccessful.

**Figure 5:**
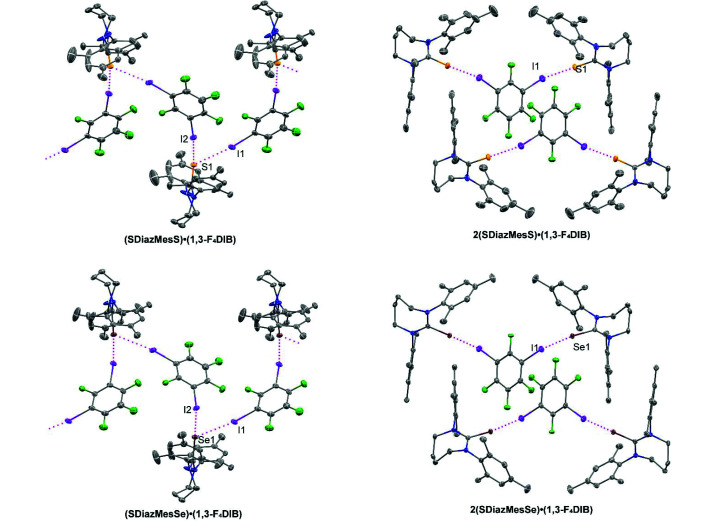
Halogen bonding in the 1,3-F_4_DIB-containing cocrystals. Intermolecular I⋯S and I⋯Se halogen bonding is indicated by magenta dotted lines. Displacement ellipsoids are drawn at the 50% probability level. Hydrogen atoms are omitted for clarity.

With 1,3-F_4_DIB as the halogen bond donor, four cocrystalline structures were obtained (Fig. 5 ▸). The first two, **(SDiazMesS)·(1,3-F_4_DIB)** and **(SDiazMesSe)·(1,3-F_4_DIB)**, are isomorphic. Both cocrsytals are obtained in the ortho­rhombic space group *P*2_1_2_1_2_1_ with one molecule of either **SDiazMesS** or **SDiazMesSe** along with one molecule of 1,3-F_4_DIB. A pair of nearly identical length C—I⋯S halogen bonds connect **SDiazMesS** or **SDiazMesSe** molecules with molecules of 1,3-F_4_DIB in alternating fashion to form helical chains propagating along the *b* axis. The packing is consolidated along the *a* axis by weak C—H⋯π interactions and in the *c* direction by C—H⋯F interactions, both involving hydrogen atoms of the heterocyclic ring. The addition of a second equivalent of **SDiazMesS** or **SDiazMesSe** results in the cocrystals **2(SDiazMesS)·(1,3-F_4_DIB)** and **2(SDiazMesSe)·­(1,3-F_4_DIB)**. Just as in the 1:1 cocrystals, the 2:1 cocrystals are isomorphic with one another, crystallizing in the monoclinic space group *C*2/*c*. Discrete halogen bonding units are formed with only one halogen bond observed at each chalcogen atom. These units stack along the *c* axis through π⋯π stacking of the 1,3-F_4_DIB rings, with ring plane-to-ring plane distances of 3.2840 (10) Å and 3.3046 (12) Å and slippage of 2.359 Å and 2.337 Å in **2(SDiazMesS)·(1,3-F_4_DIB)** and **2(SDiazMesSe)·(1,3-F_4_DIB)** respectively. These four cocrystals represent the first reported examples of halogen-bonded cocrystals of 1,3-F_4_DIB with a thio­urea or seleno­urea molecule.

**Figure 6:**
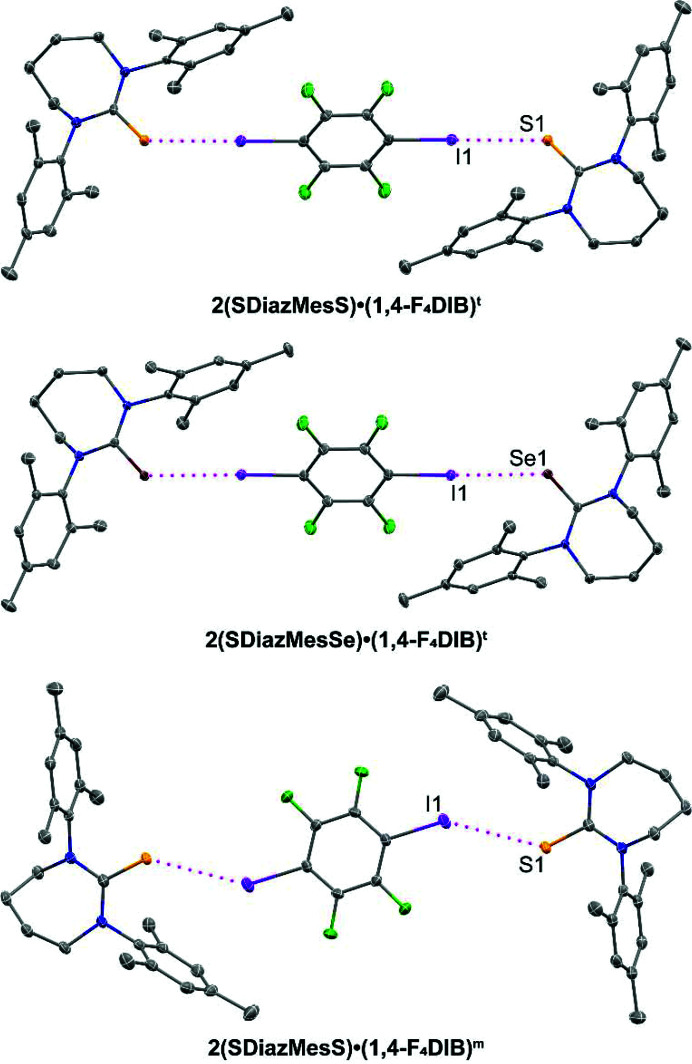
Halogen bonding in the 1,4-F_4_DIB-containing cocrystals. Intermolecular I⋯S and I⋯Se halogen bonding is indicated by magenta dotted lines. Displacement ellipsoids are drawn at the 50% probability level. Hydrogen atoms are omitted for clarity.

The reaction with the common halogen bond donor 1,4-F_4_DIB yielded two polymorphic structures with **SDiazMesS**: **2(SDiazMesS)·(1,4-F_4_DIB)^t^**, which crystallized in the triclinic space group 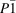 , and **2(SDiazMesS)·(1,4-F_4_DIB)^m^** which crystallized in the monoclinic space group *P*2_1_/*c*. The triclinic isomorph, **2(SDiazMesSe)·(1,4-F_4_DIB)^t^**, was obtained with **SDiazMesSe** (Fig. 6 ▸). All attempts to isolate the monoclinic isomorph with **SDiazMesSe** were unsuccessful. In all three cases, a single halogen bond is observed at each chalcogen atom, forming discrete units from two thione or selone molecules and one molecule of 1,4-F_4_DIB. For the triclinic isomorphs, the halogen-bond geometry is nearly linear, with a C—I⋯S angle of 175.67 (9)° in **2(**SDiazMesS**)·(1,4-F_4_DIB)^t^** and a C—I⋯Se angle of 173.97 (4)° in **2(**SDiazMesSe**)·(1,4-F_4_DIB)^t^**. The iodine⋯chalcogen distances in these triclinic polymorphs [I⋯S = 3.2318 (7) Å and I⋯Se = 3.2553 (3) Å] are shorter than the analogous cocrystals with thio­urea [I⋯S = 3.287 (12) Å] or seleno­urea [I⋯Se = 3.3151 (17) Å] (Arman *et al.*, 2010 ▸; Chernysheva & Haukka, 2021 ▸). Weak chalcogen⋯hydrogen interactions contribute to the stacking of these discrete units along the *a* axis. In the monoclinic polymorph, while the discrete 2:1 halogen bonding units are maintained, the C—I⋯S halogen bond is elongated relative to the triclinic polymorph, and deviates significantly from linearity [143.57 (6)°]. This geometric arrangement may suggest an intermediate between true halogen and chalcogen bonds. The (C—)I iodine atom is also involved in a weak I⋯π interaction [3.646 (2) Å]. The repositioning of the 1,4-F_4_DIB between the two **SDiazMesS** molecules in the monoclinic polymorph compared to the triclinic polymorph enable the iodine atoms to be involved in weak I⋯π interactions [3.646 (2) Å].

**Figure 7:**
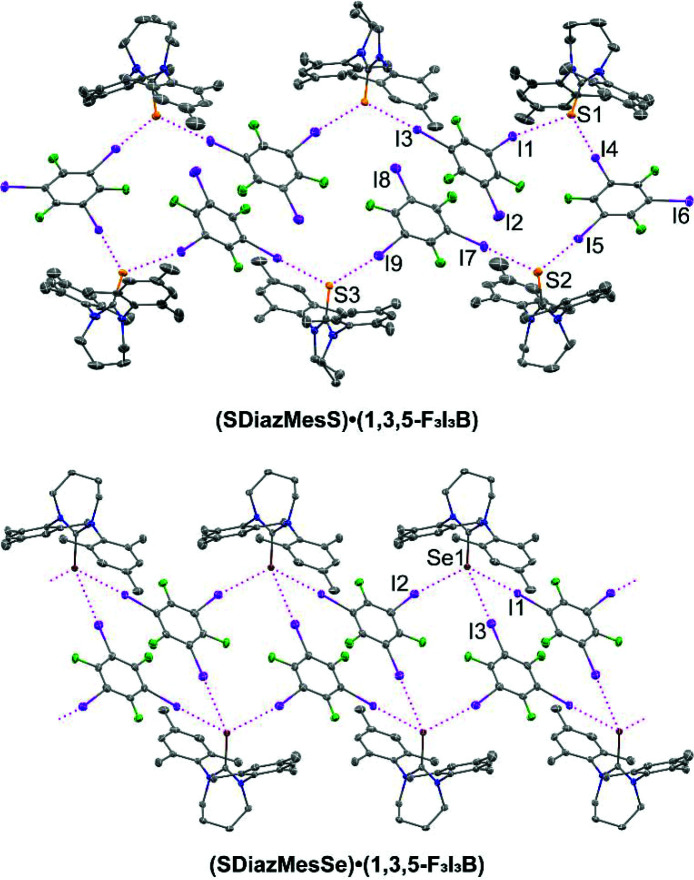
Halogen bonding in the 1,3,5-F_3_I_3_B-containing cocrystals. Intermolecular I⋯S and I⋯Se halogen bonding is indicated by magenta dotted lines. Displacement ellipsoids are drawn at the 50% probability level. Hydrogen atoms are omitted for clarity.

While the di­iodo­tetra­fluoro­benzene-containing cocrystal systems discussed thus far show roughly equivalent behavior between sulfur and selenium, the 1,3,5-F_3_I_3_B cocrystals **(SDiazMesS)·(1,3,5-F_3_I_3_B)** and **(SDiazMesSe)·(1,3,5-F_3_I_3_B)** do display a subtle, but important difference (Fig. 7 ▸). In this pair, the structures are not isomorphic, with **(SDiazMesS)·(1,3,5-F_3_I_3_B)** crystallizing in the monoclinic space group *P*2_1_/*c* and **(SDiazMesSe)·(1,3,5-F_3_I_3_B**) in space group *P*2_1_/*n*. In both cases, a pair of iodine⋯chalcogen halogen bonds are observed at each chalcogen atom. Each of these interactions ranges in normalized distance parameter, *R*_XB_, from 0.85 to 0.92. The third iodine atom of each 1,3,5-F_3_I_3_B molecule drives the differences in the overall packing motif. Of the three symmetry unique 1,3,5-F_3_I_3_B molecules in **(SDiazMesS)·(1,3,5-F_3_I_3_B)**, the third iodine atom of two of these (I2 and I8) have the appropriate geometric orientation to participate in a C—I⋯S halogen bond [C—I⋯S = 170.5 (2)° and 177.9 (2)°], but the iodine⋯sulfur distance is well beyond the sum of the van der Waals radii (*R*_XB_ = 1.12 and 1.14). This series of interactions contributes to the formation of ring-link units consisting of six **SDiazMesS** and six 1,3,5-F_3_I_3_B molecules. The third iodine atom of the final symmetry unique 1,3,5-F_3_I_3_B molecule participates in a weak type I, I⋯I interaction. In **(SDiazMesSe)·(1,3,5-F_3_I_3_B)**, two primary C—I⋯Se halogen bonds (*R*_XB_ = 0.90 and 0.92) again occur at each selenium atom. However, the third iodine atom of each 1,3,5-F_3_I_3_B molecule drives a difference in the overall packing motif. In this case, a weak C—I⋯Se halogen bond occurs roughly at the sum of the van der Waals radii (*R*_XB_ = 1.01). This weak contact, probably enabled by the increased van der Waals radius of selenium over sulfur, and therefore decreased steric congestion around the chalcogen atom, is enough to consolidate the halogen bonding motif into a ladder-like chain propagating in the *c* direction. These two cocrystals represent the first reported examples of halogen-bonded cocrystals of 1,3,5-F_3_I_3_B with a thio­urea or seleno­urea.

**Figure 8:**
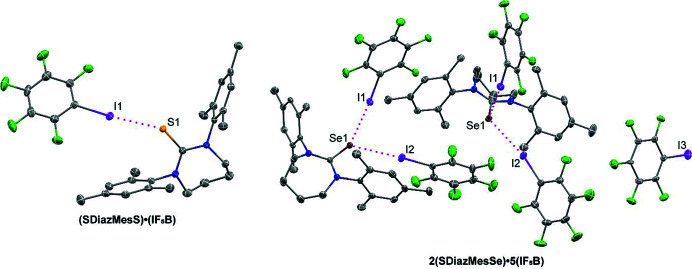
Halogen bonding in **(SDiazMesS)·(IF_5_B)** and **2(SDiazMesSe)·5(IF_5_B)**. Intermolecular I⋯S and I⋯Se halogen bonding is indicated by magenta dotted lines. Displacement ellipsoids are drawn at the 50% probability level. Hydrogen atoms are omitted for clarity.

With the halogen bond donor iodo­penta­fluoro­benzene (IF_5_B), different reaction conditions were required for **SDiazMesS** and **SDiazMesSe**, resulting in dramatically different cocrystalline structures (Fig. 8 ▸). By reacting five equivalents of IF_5_B with **SDiazMesS** in ethanol, **(SDiazMesS)·(IF_5_B)** was obtained in the orthorhombic space group *Pna*2_1_. A single I⋯S halogen bond is observed at each sulfur atom. The halogen bond length [I⋯S = 3.1809 (14) Å] is comparable to that measured in the ternary cocrystal of IF_5_B, thio­urea, and 18-crown-6 [I⋯S = 3.1977 (14) Å] (Topić & Rissanen, 2016 ▸). These halogen bonded pairs stack along the *a* axis through π⋯π stacking of the IF_5_B molecules, with a centroid to centroid distance between rings of 3.0340 (19) Å and slippage of 2.925 Å. The reaction conditions which produced **(SDiazMesS)·(IF_5_B)** did not yield the analogous cocrystal with **SDiazMesSe**. To force cocrystallization, **SDiazMesSe** was dissolved in neat IF_5_B, resulting in the cocrystal **2(SDiazMesSe)·5(IF_5_B)**. This cocrystal was obtained in the monoclinic space group *P*2_1_/*c*. In contrast to **(SDiazMesS)·(IF_5_B)**, in which only one halogen bond is observed to each chalcogen atom, two I⋯Se halogen bonds are observed to each selenium atom in **2(SDiazMesSe)·5(IF_5_B)**. The length of these halogen bonds [I⋯Se = 3.2808 (5) Å and 3.3211 (7) Å] are comparable to the analogous distance in the reported cocrystal of IF_5_B and 1,1-di­methyl­seleno­urea [I⋯Se = 3.2841 (12) Å] (Chernysheva *et al.*, 2021 ▸). The halogen bonds are similar when normalized for the increased van der Waals radius of selenium versus sulfur. The packing is consolidated along the *a* axis through weak C—F⋯π interactions.

### 3.5. Cocrystallization of **SDiazMesS** and **SDiazMesSe** with tetra­iodo­ethyl­ene

**Figure 9:**
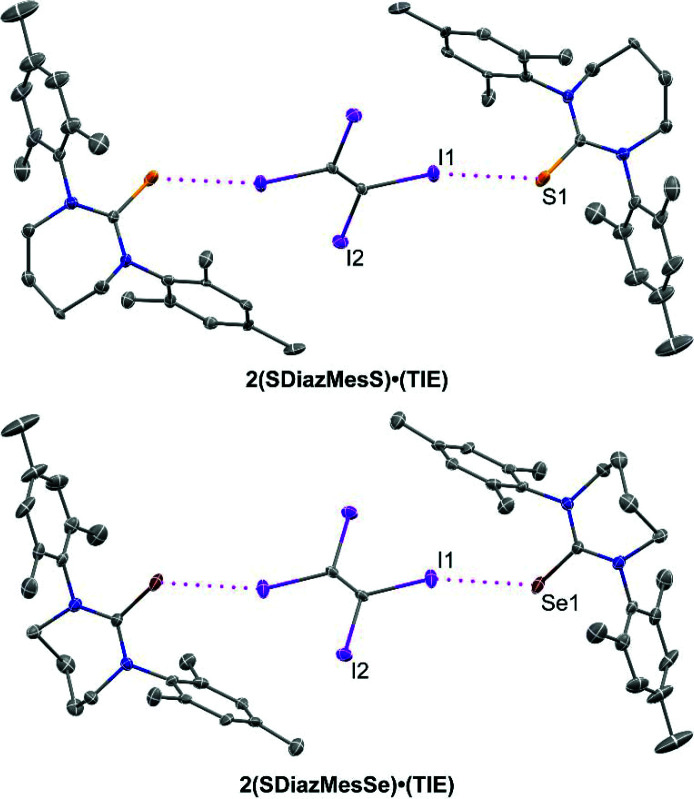
Halogen bonding in the TIE-containing cocrystals. Intermolecular I⋯S and I⋯Se halogen bonding is indicated by magenta dotted lines. Displacement ellipsoids are drawn at the 50% probability level. Hydrogen atoms are omitted for clarity.

The final pair of cocrystals, **2(SDiazMesS)·(TIE)** and **2(SDiazMesSe)·(TIE)**, are both obtained in the triclinic space group 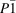 , with one thione or selone molecule and one half of a TIE molecule per asymmetric unit. A single halogen bond is observed to the chalcogen atom, contributing to discrete 2:1 units within the structure (Fig. 9 ▸). The remaining two iodine atoms of each TIE molecule appear to be pinned in place by weak C—H⋯I interactions from methyl groups of three neighboring SDiazMesE molecules, contributing to the formation of ribbons in the *bc* plane.

## 4. Conclusions

The heterocyclic molecules hexa­hydro-1,3-bis­(2,4,6-tri­methyl­phenyl)-2*H*-1,3-diazepine-2-thione and hexa­hydro-1,3-bis­(2,4,6-tri­methyl­phenyl)-2*H*-1,3-diazepine-2-selone provided a robust template for halogen and/or chalcogen bonding interactions, yielding a total of 24 new cocrystal structures. The reaction with molecule diiodine provided products incorporating S—I—I and Se—I—I fragments with a wide range of bond orders. When this reaction was conducted in acetone, oxidative addition of acetone to the chalcogen atom allowed the formation of new C—S, C—Se and C—C covalent bonds under mild conditions. Cocrystallization with iodo­penta­fluoro­benzene, 1,2-, 1,3- and 1,4-di­iodo­tetra­fluoro­benzene, 1,3,5-tri­fluoro­tri­iodo­benzene, and tetra­iodo­ethyl­ene reveals structures that, in most cases, show a preference for halogen over chalcogen bonding and are typically isomorphic. This series of structural data supports the power of crystallographic study to reveal unexpected and unique interactions and reaction pathways.

## Supplementary Material

Crystal structure: contains datablock(s) SDiazMesS, SDiazMesSe, SDiazMesS_MeCN, SDiazMesSe_MeCN, SDiazMesSI2, SDiazMesSeI2, SDiazMesSI2_I2, SDiazMesSeI_I3, SDiazMesSeI_I3_DCM, SDiazMesSeDMK_I3_I2, SDiazMesSMIBK_I3, SDiazMesSeMIBK_I3, SDiazMesS_12F4DIB, SDiazMesS_13F4DIB, SDiazMesSe_13F4DIB, 2SDiazMesS_13F4DIB, 2SDiazMesSe_13F4DIB, 2SDiazMesS_14F4DIB_t, 2SDiazMesSe_14F4DIB_t, 2SDiazMesS_14F4DIB_m, SDiazMesS_135F3I3B, SDiazMesSe_135F3I3B, SDiazMesS_IF5B, 2SDiazMesSe_5F5IB, 2SDiazMesS_TIE, 2SDiazMesSe_TIE. DOI: 10.1107/S2052520622008150/rm5061sup1.cif

Structure factors: contains datablock(s) SDiazMesS. DOI: 10.1107/S2052520622008150/rm5061SDiazMesSsup2.hkl

Structure factors: contains datablock(s) SDiazMesSe. DOI: 10.1107/S2052520622008150/rm5061SDiazMesSesup3.hkl

Structure factors: contains datablock(s) SDiazMesS_MeCN. DOI: 10.1107/S2052520622008150/rm5061SDiazMesS_MeCNsup4.hkl

Structure factors: contains datablock(s) SDiazMesSe_MeCN. DOI: 10.1107/S2052520622008150/rm5061SDiazMesSe_MeCNsup5.hkl

Structure factors: contains datablock(s) SDiazMesSI2. DOI: 10.1107/S2052520622008150/rm5061SDiazMesSI2sup6.hkl

Structure factors: contains datablock(s) SDiazMesSeI2. DOI: 10.1107/S2052520622008150/rm5061SDiazMesSeI2sup7.hkl

Structure factors: contains datablock(s) SDiazMesSI2_I2. DOI: 10.1107/S2052520622008150/rm5061SDiazMesSI2_I2sup8.hkl

Structure factors: contains datablock(s) SDiazMesSeI_I3. DOI: 10.1107/S2052520622008150/rm5061SDiazMesSeI_I3sup9.hkl

Structure factors: contains datablock(s) SDiazMesSeI_I3_DCM. DOI: 10.1107/S2052520622008150/rm5061SDiazMesSeI_I3_DCMsup10.hkl

Structure factors: contains datablock(s) SDiazMesSeDMK_I3_I2. DOI: 10.1107/S2052520622008150/rm5061SDiazMesSeDMK_I3_I2sup11.hkl

Structure factors: contains datablock(s) SDiazMesSMIBK_I3. DOI: 10.1107/S2052520622008150/rm5061SDiazMesSMIBK_I3sup12.hkl

Structure factors: contains datablock(s) SDiazMesSeMIBK_I3. DOI: 10.1107/S2052520622008150/rm5061SDiazMesSeMIBK_I3sup13.hkl

Structure factors: contains datablock(s) SDiazMesS_12F4DIB. DOI: 10.1107/S2052520622008150/rm5061SDiazMesS_12F4DIBsup14.hkl

Structure factors: contains datablock(s) SDiazMesS_13F4DIB. DOI: 10.1107/S2052520622008150/rm5061SDiazMesS_13F4DIBsup15.hkl

Structure factors: contains datablock(s) SDiazMesSe_13F4DIB. DOI: 10.1107/S2052520622008150/rm5061SDiazMesSe_13F4DIBsup16.hkl

Structure factors: contains datablock(s) 2SDiazMesS_13F4DIB. DOI: 10.1107/S2052520622008150/rm50612SDiazMesS_13F4DIBsup17.hkl

Structure factors: contains datablock(s) 2SDiazMesSe_13F4DIB. DOI: 10.1107/S2052520622008150/rm50612SDiazMesSe_13F4DIBsup18.hkl

Structure factors: contains datablock(s) 2SDiazMesS_14F4DIB_t. DOI: 10.1107/S2052520622008150/rm50612SDiazMesS_14F4DIB_tsup19.hkl

Structure factors: contains datablock(s) 2SDiazMesSe_14F4DIB_t. DOI: 10.1107/S2052520622008150/rm50612SDiazMesSe_14F4DIB_tsup20.hkl

Structure factors: contains datablock(s) 2SDiazMesS_14F4DIB_m. DOI: 10.1107/S2052520622008150/rm50612SDiazMesS_14F4DIB_msup21.hkl

Structure factors: contains datablock(s) SDiazMesS_135F3I3B. DOI: 10.1107/S2052520622008150/rm5061SDiazMesS_135F3I3Bsup22.hkl

Structure factors: contains datablock(s) SDiazMesSe_135F3I3B. DOI: 10.1107/S2052520622008150/rm5061SDiazMesSe_135F3I3Bsup23.hkl

Structure factors: contains datablock(s) SDiazMesS_IF5B. DOI: 10.1107/S2052520622008150/rm5061SDiazMesS_IF5Bsup24.hkl

Structure factors: contains datablock(s) 2SDiazMesSe_5F5IB. DOI: 10.1107/S2052520622008150/rm50612SDiazMesSe_5F5IBsup25.hkl

Structure factors: contains datablock(s) 2SDiazMesS_TIE. DOI: 10.1107/S2052520622008150/rm50612SDiazMesS_TIEsup26.hkl

Structure factors: contains datablock(s) 2SDiazMesSe_TIE. DOI: 10.1107/S2052520622008150/rm50612SDiazMesSe_TIEsup27.hkl

Tables S1-S24. DOI: 10.1107/S2052520622008150/rm5061sup28.pdf

CCDC references: 2201575, 2201576, 2201577, 2201578, 2201579, 2201580, 2201581, 2201582, 2201583, 2201584, 2201585, 2201586, 2201587, 2201588

## Figures and Tables

**Table 1:** Selected bond lengths (Å) in **SDiazMes*E***, **(SDiazMes*E*)·(MeCN)** (*E* = S, Se) and the products of reaction with I_2_

Compound	*d*_C=*E*_	*d*_C—N_		*E*—I	I—I
**SDiazMesS**	1.6887 (13)	1.3625 (16)	1.361 (2)	–	–
**SDiazMesSe**	1.851 (2)	1.361 (3)	1.348 (3)	–	–
	1.850 (2)	1.356 (3)	1.353 (3)	–	–
**(SDiazMesS)·(MeCN)**	1.6821 (15)	1.3627 (17)	1.3689 (15)	–	–
**(SDiazMesSe)·(MeCN)**	1.8498 (18)	1.355 (2)	1.3586 (19)	–	–
**SDiazMesS—I_2_**	1.738 (3)	1.356 (4)	1.337 (5)	2.6738 (9)	2.8794 (4)
**SDiazMesSe—I_2_**	1.8973 (16)	1.334 (3)	1.351 (2)	2.7559 (4)	2.9106 (4)
**(SDiazMesS—I_2_)·(I_2_)**	1.7555 (18)	1.335 (3)	1.344 (2)	2.5052 (5)	3.0803 (4)
**[(SDiazMesSe—I)(I_3_)]**	1.921 (10)	1.338 (13)	1.339 (13)	2.5807 (14)	3.2052 (10)
**[(SDiazMesSe—I)(I_3_)]·(DCM)**	1.9274 (17)	1.331 (3)	1.338 (3)	2.5958 (3)	3.1867 (3)
**[(SDiazMesSe-DMK)(I_3_)]·(I_2_)**	1.910 (9)	1.320 (12)	1.336 (11)	–	–
**[(SDiazMesS-MIBK)(I_3_)]**	1.772 (4)	1.339 (4)	1.329 (4)	–	–
**[(SDiazMesSe-MIBK)(I_3_)]**	1.9284 (17)	1.340 (3)	1.328 (2)	–	–
