# Supplementary material for: Heterocyclic 1,3-diazepine-based thio­nes and selones as versatile halogen-bond acceptors
Source: Acta Crystallogr B Struct Sci Cryst Eng Mater. 2022 Aug 31;78(Pt 5):745–55. doi: 10.1107/S2052520622008150 (PMC9533313; doi:10.1107/S2052520622008150)
Supplement: Supplementary file 28 [file b-78-00745-sup28.pdf]

Acta Crystallographica Section B research papers

Heterocyclic 1,3-diazepine-based thiones and selones as versatile halogen bond acceptors

Arianna C. Ragusa[a], Andrew J. Peloquin[a], Marjan M. Shahani[b], Keri N. Dowling[b], James A. Golen[c], Colin D. McMillen[a], Daniel Rabinovich[bd] and William T. Pennington[a*]

[a]Department of Chemistry, Clemson University, 219 Hunter Laboratories, Clemson, SC, 29634, USA, [b]Department of Chemistry, University of North Carolina at Charlotte, 9201 University City Blvd, Charlotte, NC, 28223, USA, [c]Department of Chemistry and Biochemistry, University of Massachusetts Dartmouth, North Dartmouth, MA, 02747, USA, and [d]Joint School of Nanoscience and Nanoengineering, 2907 E. Gate City Blvd, Greensboro, NC, 27401, USA

Correspondence email: billp@clemson.edu

Funding information Air Force Institute of Technology (doi:100009924) (scholarship to Andrew J. Peloquin); National Science Foundation (doi:100000001) (grant No. CHE-1560300; grant No. CHE-2050042).

Table S1 Halogen bond geometries in organoiodine-containing cocrystals (Å, °)

Compound		$d_{I\cdots E}$	R_{XB}[i]	$\theta_{C–I\cdots E}$	$\theta_{C=E\cdots I}$	$\theta_1 - \theta_2$[ii]
(SDiazMesS)•(1,2-F_4DIB)	I1···S1	3.2092(12)	0.85	173.36(13)	129.75(16)	43.6(3)
(SDiazMesS)•(1,3-F_4DIB)	I1···S1	3.2795(15)	0.87	170.80(15)	120.09(19)	50.7(3)
	I2···S1	3.3176(15)	0.88	170.23(15)	133.2(2)	37.0(4)
(SDiazMesSe)•(1,3-F_4DIB)	I1···Se1	3.3348(5)	0.86	170.03(10)	115.89(12)	54.1(2)
	I2···Se1	3.3550(7)	0.87	166.71(10)	131.03(12)	35.7(2)

IMPORTANT: this document contains embedded data - to preserve data integrity, please ensure where possible that the IUCr Word tools (available from http://journals.iucr.org/services/docxtemplate/) are installed when editing this document.

2(SDiazMesS)•(1,3-F_4DIB)	I1···S1	3.3131(11)	0.88	167.09(8)	123.63(10)	43.46(18)
2(SDiazMesSe)•(1,3-F_4DIB)	I1···Se1	3.3280(11)	0.86	168.37(10)	119.78(11)	48.5(2)
(SDiazMesS)•(1,4-F_4DIB)^t	I1···S1	3.2318(7)	0.85	175.67(9)	123.21(9)	52.46(18)
(SDiazMesS)•(1,4-F_4DIB)^m	I1···S1	3.4491(5)	0.91	143.57(6)	131.44(8)	12.13(14)
(SDiazMesSe)•(1,4-F_4DIB)^t	I1···Se1	3.2553(3)	0.84	173.97(4)	120.80(4)	53.17(8)
(SDiazMesS)•(1,3,5-F_3I_3B)	I1···S1	3.434(2)	0.91	156.0(3)	127.8(3)	28.2(3)
	I4···S1	3.236(2)	0.86	175.8(2)	127.1(3)	48.7(5)
	I5···S2	3.213(2)	0.85	175.9(2)	111.6(3)	64.3(5)
	I7···S2	3.445(2)	0.91	165.5(2)	121.5(3)	44.0(5)
	I3···S3	3.369(2)	0.89	170.7(2)	110.9(3)	59.8(5)
	I9···S2	3.4317(19)	0.91	171.7(3)	118.5(3)	53.2(6)
(SDiazMesSe)•(1,3,5-F_3I_3B)	I1···Se1	3.5635(5)	0.92	164.91(12)	110.50(12)	54.4(2)
	I2···Se1	3.4758(5)	0.90	170.07(11)	116.97(12)	53.1(2)
	I3···Se1	3.9070(8)	1.01	176.73(10)	131.29(11)	45.4(2)
(SDiazMesS)•(IF_5B)	I1···S1	3.1809(14)	0.84	173.11(13)	131.18(16)	41.9(3)
2(SDiazMesSe)•5(IF_5B)	I1···Se1	3.2808(5)	0.85	175.12(8)	108.01(6)	67.11(14)
	I2···Se1	3.3211(7)	0.86	170.52(7)	129.54(9)	40.98(16)
2(SDiazMesS)•(TIE)	I1···S1	3.1969(13)	0.85	164.79(11)	131.12(11)	33.7(2)
2(SDiazMesSe)•(TIE)	I1···Se1	3.2139(4)	0.83	162.73(15)	129.35(8)	33.4(2)

(i)$R_{XB} = d_{X\cdots Y}/\sum d_{Vdw}$, the ratio of the distance between the donor atom (i.e., I) and the acceptor atom (i.e., S, Se) to the sum of their van der Waals radii (S, 1.80 Å; Se, 1.90 Å; I, 1.98 Å). (ii)$\theta_1 - \theta_2 = |[(\theta_{C\text{–}I\cdots E}) - (\theta_{C=E\cdots I})]|$

Table S2 Experimental details

	(SDiazMesS)	(SDiazMesSe)	(SDiazMesS_MeCN)	(SDiazMesSe_MeCN)
Crystal data				
Chemical formula	$C_{23}H_{30}N_2S$	$C_{23}H_{30}N_2Se$	$C_{23}H_{30}N_2S\cdot C_2H_3N$	$C_{23}H_{30}N_2Se\cdot C_2H_3N$
M_r	366.55	413.45	407.60	454.50
Crystal system, space group	Monoclinic, $P2_1/c$	Orthorhombic, *Pbca*	Monoclinic, $P2_1/c$	Monoclinic, $P2_1/c$
Temperature (K)	100	100	100	100
a, *b*, *c* (Å)	16.4064 (14), 8.7386 (9), 14.8229 (14)	17.4279 (4), 15.2522 (4), 31.0253 (7)	13.0884 (6), 12.9760 (5), 14.4293 (7)	13.1365 (3), 13.3024 (2), 14.2478 (3)
α, β, γ (°)	90, 104.983 (3), 90	90, 90, 90	90, 113.028 (2), 90	90, 113.1557 (7), 90
V ($Å^3$)	2052.9 (3)	8247.0 (3)	2255.32 (18)	2289.18 (8)
Z	4	16	4	4
Radiation type	Mo *K*α	Mo *K*α	Mo *K*α	Mo *K*α
μ (mm^{-1})	0.17	1.83	0.16	1.66
Crystal size (mm)	0.30 × 0.19 × 0.17	0.08 × 0.07 × 0.07	0.26 × 0.17 × 0.10	0.23 × 0.19 × 0.10
Data collection				
Diffractometer	Bruker D8 Venture Photon 2	Bruker D8 Venture Photon 2	Bruker D8 Venture Photon 2	Bruker D8 Venture Photon 2
Absorption correction	Multi-scan *SADABS* v2016/2	Multi-scan *SADABS* v2016/2 (Bruker AXS Inc., 2017)	Multi-scan *SADABS* v2016/2 (Bruker AXS Inc., 2017)	Multi-scan *SADABS* v2016/2 (Bruker AXS Inc., 2017)

	(Bruker AXS Inc., 2017)			
T_{min}, T_{max}	0.706, 0.746	0.697, 0.746	0.711, 0.746	0.683, 0.746
No. of measured, independent and observed [$I > 2\sigma(I)$] reflections	33473, 4240, 3717	72880, 9109, 7294	34671, 5148, 4419	40871, 5251, 4637
R_{int}	0.044	0.072	0.046	0.046
(sin θ/λ)$_{max}$ (Å^{-1})	0.628	0.642	0.649	0.650
Refinement				
$R[F^2 > 2\sigma(F^2)]$, $wR(F^2)$, S	0.037, 0.097, 1.03	0.036, 0.078, 1.03	0.038, 0.096, 1.06	0.026, 0.061, 1.08
No. of reflections	4240	9109	5148	5251
No. of parameters	241	481	288	288
No. of restraints	0	0	36	36
H-atom treatment	H-atom parameters constrained	H-atom parameters constrained	H-atom parameters constrained	H-atom parameters constrained
	$w = 1/[\sigma^2(F_o^2) + (0.0371P)^2 + 1.5577P]$ where $P = (F_o^2 + 2F_c^2)/3$	$w = 1/[\sigma^2(F_o^2) + (0.0255P)^2 + 7.8329P]$ where $P = (F_o^2 + 2F_c^2)/3$	$w = 1/[\sigma^2(F_o^2) + (0.0294P)^2 + 1.5344P]$ where $P = (F_o^2 + 2F_c^2)/3$	$w = 1/[\sigma^2(F_o^2) + (0.0125P)^2 + 1.9573P]$ where $P = (F_o^2 + 2F_c^2)/3$
$\Delta\rho_{max}$, $\Delta\rho_{min}$ (e Å^{-3})	0.37, −0.27	0.39, −0.33	0.28, −0.21	0.36, −0.33

Absolute structure	-	-	-	-
Absolute structure parameter	-	-	-	-

	(SDiazMesSI2)	(SDiazMesSeI2)	(SDiazMesSI2_I2)	(SDiazMesSeI_I3)
Crystal data				
Chemical formula	$C_{23}H_{30}I_2N_2S$	$C_{23}H_{30}I_2N_2Se$	$2(C_{23}H_{30}I_2N_2S)\cdot 2(I_2)$	$C_{23}H_{30}IN_2Se\cdot I_3$
M_r	620.35	667.25	1748.30	921.05
Crystal system, space group	Triclinic, *P*-1	Triclinic, *P*-1	Triclinic, *P*-1	Monoclinic, $P2_1/n$
Temperature (K)	100	100	100	100
a, *b*, *c* (Å)	7.9505 (4), 8.5048 (4), 18.2812 (9)	8.0282 (6), 8.4305 (8), 18.3525 (16)	8.3022 (9), 11.3510 (11), 15.5656 (16)	11.0063 (7), 22.9632 (14), 11.1015 (6)
α, β, γ (°)	81.040 (3), 89.926 (2), 85.125 (2)	81.814 (4), 89.706 (4), 85.171 (4)	83.691 (4), 87.180 (4), 75.024 (4)	90, 93.024 (2), 90
V ($Å^3$)	1216.54 (10)	1225.08 (18)	1408.1 (3)	2801.9 (3)
Z	2	2	1	4
Radiation type	Mo *K*α	Mo *K*α	Mo *K*α	Mo *K*α
μ (mm^{-1})	2.68	4.06	4.51	5.76
Crystal size (mm)	0.17 × 0.12 × 0.08	0.19 × 0.15 × 0.13	0.19 × 0.09 × 0.04	0.06 × 0.06 × 0.02
Data collection				

Diffractometer	Bruker D8 Venture Photon 2	Bruker D8 Venture Photon 2	Bruker D8 Venture Photon 2	Bruker D8 Venture Photon 2
Absorption correction	Multi-scan *SADABS* v2016/2 (Bruker AXS Inc., 2017)	Multi-scan *SADABS* v2016/2 (Bruker AXS Inc., 2017)	Multi-scan *SADABS* v2016/2 (Bruker AXS Inc., 2017)	Multi-scan *SADABS* v2016/2 (Bruker AXS Inc., 2017)
T_{min}, T_{max}	0.636, 0.746	0.634, 0.746	0.579, 0.746	0.606, 0.746
No. of measured, independent and observed [$I > 2\sigma(I)$] reflections	37544, 5455, 4834	53696, 7479, 6504	71058, 8260, 7603	36709, 6428, 5536
R_{int}	0.067	0.045	0.035	0.091
$(\sin \theta/\lambda)_{max}$ (Å^{-1})	0.646	0.715	0.706	0.650
Refinement				
$R[F^2 > 2\sigma(F^2)]$, $wR(F^2)$, S	0.034, 0.072, 1.12	0.023, 0.049, 1.10	0.017, 0.039, 1.18	0.062, 0.127, 1.17
No. of reflections	5455	7479	8260	6428
No. of parameters	259	259	277	278
No. of restraints	0	0	0	30
H-atom treatment	H-atom parameters constrained	H-atom parameters constrained	H-atom parameters constrained	H-atom parameters constrained
	$w = 1/[\sigma^2(F_o^2) + (0.0141P)^2 + 2.7069P]$	$w = 1/[\sigma^2(F_o^2) + (0.0078P)^2 + 1.5207P]$	$w = 1/[\sigma^2(F_o^2) + (0.0082P)^2 + 1.3172P]$ where $P = (F_o^2 + 2F_c^2)/3$	$w = 1/[\sigma^2(F_o^2) + (0.0131P)^2 + 65.9822P]$

	where $P = (F_o^2 + 2F_c^2)/3$	where $P = (F_o^2 + 2F_c^2)/3$		where $P = (F_o^2 + 2F_c^2)/3$
$\Delta\rho_{max}$, $\Delta\rho_{min}$ (e Å^{-3})	1.35, −1.40	0.70, −0.75	0.50, −0.74	2.17, −1.40
Absolute structure	-	-	-	-
Absolute structure parameter	-	-	-	-

	(SDiazMesSeI_I3_DCM)	(SDiazMesSeDMK_I3_I2)	(SDiazMesSMIBK_I3)	(SDiazMesSeMIBK_I3)
Crystal data				
Chemical formula	$C_{23}H_{30}IN_2Se \cdot I_3 \cdot CH_2Cl_2$	$3.333(I_{1.5}) \cdot C_{26}H_{35}N_2OSe$	$I_3 \cdot C_{29}H_{41}N_2OS$	$I_3 \cdot C_{29}H_{41}N_2OSe$
M_r	1005.97	1105.02	846.40	893.30
Crystal system, space group	Triclinic, *P*-1	Monoclinic, *C*2/*c*	Triclinic, *P*-1	Triclinic, *P*-1
Temperature (K)	100	100	100	100
a, *b*, *c* (Å)	8.4541 (4), 8.5353 (4), 22.0045 (11)	13.2501 (6), 11.0708 (6), 45.509 (3)	8.7838 (8), 14.2194 (15), 15.1418 (15)	8.8091 (5), 14.2584 (8), 15.1228 (9)
α, β, γ (°)	93.258 (2), 90.198 (2), 98.032 (2)	90, 96.722 (2), 90	63.522 (3), 74.494 (4), 80.494 (4)	63.952 (2), 74.061 (2), 81.113 (2)
V (Å^3)	1569.58 (13)	6629.7 (6)	1628.9 (3)	1639.67 (17)
Z	2	8	2	2
Radiation type	Mo *K*α	Mo *K*α	Mo *K*α	Mo *K*α

μ (mm^{-1})	5.32	5.81	2.97	3.99
Crystal size (mm)	0.24 × 0.15 × 0.05	0.17 × 0.15 × 0.05	0.11 × 0.09 × 0.08	0.17 × 0.11 × 0.08
Data collection				
Diffractometer	Bruker D8 Venture Photon 2	Bruker D8 Venture Photon 2	Bruker D8 Venture Photon 2	Bruker D8 Venture Photon 2
Absorption correction	Multi-scan *SADABS* v2016/2 (Bruker AXS Inc., 2017)	Multi-scan *SADABS* v2016/2 (Bruker AXS Inc., 2017)	Multi-scan *SADABS* v2016/2 (Bruker AXS Inc., 2017)	Multi-scan *SADABS* v2016/2 (Bruker AXS Inc., 2017)
T_{min}, T_{max}	0.527, 0.746	0.561, 0.745	0.645, 0.746	0.640, 0.746
No. of measured, independent and observed [$I > 2\sigma(I)$] reflections	64752, 8821, 7994	46614, 6580, 5737	60178, 8827, 6624	75078, 9229, 7989
R_{int}	0.038	0.041	0.052	0.041
$(\sin \theta/\lambda)_{max}$ (Å^{-1})	0.695	0.620	0.687	0.696
Refinement				
$R[F^2 > 2\sigma(F^2)]$, $wR(F^2)$, S	0.019, 0.043, 1.13	0.067, 0.142, 1.10	0.033, 0.069, 1.20	0.024, 0.052, 1.14
No. of reflections	8821	6580	8827	9229
No. of parameters	304	325	333	333

No. of restraints	0	0	0	0
H-atom treatment	H-atom parameters constrained	H-atom parameters constrained	H-atom parameters constrained	H-atom parameters constrained
	$w = 1/[\sigma^2(F_o^2) + (0.0077P)^2 + 1.7629P]$ where $P = (F_o^2 + 2F_c^2)/3$	$w = 1/[\sigma^2(F_o^2) + (0.018P)^2 + 376.8008P]$ where $P = (F_o^2 + 2F_c^2)/3$	$w = 1/[\sigma^2(F_o^2) + 4.6197P]$ where $P = (F_o^2 + 2F_c^2)/3$	$w = 1/[\sigma^2(F_o^2) + (0.0083P)^2 + 2.5037P]$ where $P = (F_o^2 + 2F_c^2)/3$
$\Delta\rho_{max}$, $\Delta\rho_{min}$ (e Å^{-3})	0.81, −0.88	4.07, −3.13	2.75, −0.92	1.18, −1.66
Absolute structure	-?	-	-	-
Absolute structure parameter	-	-	-	-

	(SDiazMesS_12F4DIB)	(SDiazMesS_13F4DIB)	(SDiazMesSe_13F4DIB)	(2SDiazMesS_13F4DIB)
Crystal data				
Chemical formula	$C_6F_4I_2\cdot C_{23}H_{30}N_2S$	$C_6F_4I_2\cdot C_{23}H_{30}N_2S$	$C_6F_4I_2\cdot C_{23}H_{30}N_2Se$	$C_6F_4I_2\cdot 2(C_{23}H_{30}N_2S)$
M_r	768.41	768.41	815.31	1134.96
Crystal system, space group	Orthorhombic, $Pna2_1$	Orthorhombic, $P2_12_12_1$	Orthorhombic, $P2_12_12_1$	Monoclinic, $C2/c$
Temperature (K)	100	100	100	100

a, *b*, *c* (Å)	7.6668 (5), 16.8546 (11), 22.3786 (15)	8.2080 (2), 12.9974 (2), 27.2463 (5)	8.2583 (2), 12.9953 (4), 27.2240 (7)	39.0981 (14), 17.0474 (6), 7.3957 (2)
α, β, γ (°)	90, 90, 90	90, 90, 90	90, 90, 90	90, 94.344 (1), 90
V ($Å^3$)	2891.8 (3)	2906.71 (10)	2921.66 (14)	4915.2 (3)
Z	4	4	4	4
Radiation type	Mo *K*α	Mo *K*α	Mo *K*α	Mo *K*α
μ (mm^{-1})	2.30	2.28	3.45	1.42
Crystal size (mm)	0.22 × 0.07 × 0.07	0.15 × 0.14 × 0.06	0.29 × 0.08 × 0.07	0.28 × 0.09 × 0.03
Data collection				
Diffractometer	Bruker D8 Venture Photon 2	Bruker D8 Venture Photon 2	Bruker D8 Venture Photon 2	Bruker D8 Venture Photon 2
Absorption correction	Multi-scan *SADABS* v2016/2 (Bruker AXS Inc., 2017)	Multi-scan *SADABS* v2016/2 (Bruker AXS Inc., 2017)	Multi-scan *SADABS* v2016/2 (Bruker AXS Inc., 2017)	Multi-scan *SADABS* v2016/2 (Bruker AXS Inc., 2017)
T_{min}, T_{max}	0.621, 0.746	0.676, 0.746	0.494, 0.746	0.663, 0.746
No. of measured, independent and observed [$I > 2\sigma(I)$] reflections	50096, 7186, 6786	28002, 6938, 6327	85940, 7878, 7491	40822, 5102, 4240
R_{int}	0.044	0.048	0.047	0.060
$(\sin \theta/\lambda)_{max}$ ($Å^{-1}$)	0.668	0.658	0.686	0.628

Refinement				
$R[F^2 > 2\sigma(F^2)]$, $wR(F^2)$, S	0.025, 0.058, 1.10	0.031, 0.065, 1.09	0.022, 0.044, 1.13	0.029, 0.061, 1.16
No. of reflections	7186	6938	7878	5102
No. of parameters	349	386	386	334
No. of restraints	1	96	96	0
H-atom treatment	H-atom parameters constrained	H-atom parameters constrained	H-atom parameters constrained	H-atom parameters constrained
	$w = 1/[\sigma^2(F_o^2) + (0.0178P)^2 + 2.9992P]$ where $P = (F_o^2 + 2F_c^2)/3$	$w = 1/[\sigma^2(F_o^2) + 4.5084P]$ where $P = (F_o^2 + 2F_c^2)/3$	$w = 1/[\sigma^2(F_o^2) + (0.003P)^2 + 3.3501P]$ where $P = (F_o^2 + 2F_c^2)/3$	$w = 1/[\sigma^2(F_o^2) + 15.3992P]$ where $P = (F_o^2 + 2F_c^2)/3$
$\Delta\rho_{max}$, $\Delta\rho_{min}$ (e Å^{-3})	0.77, −0.70	1.19, −1.17	0.99, −0.93	1.02, −0.53
Absolute structure	Flack x determined using 3124 quotients [(I+)-(I-)]/[(I+)+(I-)] (Parsons, Flack and Wagner, Acta Cryst. B69 (2013) 249-259).	Flack x determined using 2576 quotients [(I+)-(I-)]/[(I+)+(I-)] (Parsons, Flack and Wagner, Acta Cryst. B69 (2013) 249-259).	Flack x determined using 3173 quotients [(I+)-(I-)]/[(I+)+(I-)] (Parsons, Flack and Wagner, Acta Cryst. B69 (2013) 249-259).	-
Absolute structure parameter	−0.025 (7)	−0.034 (12)	0.003 (4)	-

	(2SDiazMesSe_13F4DIB)	(2SDiazMesS_14F4DIB_t)	(2SDiazMesSe_14F4DIB_t)	(2SDiazMesS_14F4DIB_m)
Crystal data				
Chemical formula	$C_6F_4I_2 \cdot 2(C_{23}H_{30}N_2Se)$	$C_6F_4I_2 \cdot 2(C_{23}H_{30}N_2S)$	$C_6F_4I_2 \cdot 2(C_{23}H_{30}N_2Se)$	$C_6F_4I_2 \cdot 2(C_{23}H_{30}N_2S)$
M_r	1228.76	1134.96	1228.76	1134.96
Crystal system, space group	Monoclinic, *C*2/*c*	Triclinic, *P*-1	Triclinic, *P*-1	Monoclinic, $P2_1/c$
Temperature (K)	100	100	100	100
a, *b*, *c* (Å)	39.058 (4), 17.2429 (14), 7.4287 (6)	7.5020 (7), 12.2475 (11), 14.4780 (13)	7.5969 (4), 12.1995 (7), 14.4439 (8)	13.3377 (6), 15.2129 (6), 13.0613 (5)
α, β, γ (°)	90, 94.012 (4), 90	104.905 (3), 95.822 (3), 104.609 (3)	104.6128 (18), 95.7464 (19), 104.1188 (18)	90, 109.998 (1), 90
V (Å^3)	4990.8 (7)	1224.10 (19)	1237.42 (12)	2490.41 (18)
Z	4	1	1	2
Radiation type	Mo *K*α	Mo *K*α	Mo *K*α	Mo *K*α
μ (mm^{-1})	2.77	1.43	2.80	1.40
Crystal size (mm)	0.39 × 0.04 × 0.04	0.18 × 0.04 × 0.04	0.21 × 0.17 × 0.10	0.18 × 0.16 × 0.06
Data collection				
Diffractometer	Bruker D8 Venture Photon 2	Bruker D8 Venture Photon 2	Bruker D8 Venture Photon 2	Bruker D8 Venture Photon 2

Absorption correction	Multi-scan *SADABS* v2016/2 (Bruker AXS Inc., 2017)	Multi-scan *SADABS* v2016/2 (Bruker AXS Inc., 2017)	Multi-scan *SADABS* v2016/2 (Bruker AXS Inc., 2017)	Multi-scan *SADABS* v2016/2 (Bruker AXS Inc., 2017)
T_{min}, T_{max}	0.623, 0.745	0.679, 0.746	0.582, 0.746	0.683, 0.746
No. of measured, independent and observed [$I > 2\sigma(I)$] reflections	52044, 4907, 4119	16839, 5058, 4439	42081, 7215, 6861	53110, 5713, 5146
R_{int}	0.068	0.045	0.031	0.042
$(\sin \theta/\lambda)_{max}$ (Å^{-1})	0.617	0.628	0.705	0.650
Refinement				
$R[F^2 > 2\sigma(F^2)]$, $wR(F^2)$, S	0.031, 0.068, 1.24	0.030, 0.060, 1.13	0.017, 0.041, 1.07	0.024, 0.056, 1.10
No. of reflections	4907	5058	7215	5713
No. of parameters	297	295	295	295
No. of restraints	0	0	0	0
H-atom treatment	H-atom parameters constrained	H-atom parameters constrained	H-atom parameters constrained	H-atom parameters constrained
	$w = 1/[\sigma^2(F_o^2) + 28.6286P]$	$w = 1/[\sigma^2(F_o^2) + (0.0038P)^2 + 1.3971P]$	$w = 1/[\sigma^2(F_o^2) + (0.0149P)^2 + 0.707P]$	$w = 1/[\sigma^2(F_o^2) + (0.0119P)^2 + 3.5016P]$

	where $P = (F_o^2 + 2F_c^2)/3$	where $P = (F_o^2 + 2F_c^2)/3$	where $P = (F_o^2 + 2F_c^2)/3$	where $P = (F_o^2 + 2F_c^2)/3$
$\Delta\rho_{max}$, $\Delta\rho_{min}$ (e Å^{-3})	1.00, −0.67	1.15, −0.66	0.44, −0.51	1.26, −1.41
Absolute structure	-	-	-	-
Absolute structure parameter	-	-	-	-

	(SDiazMesS_135F3I3B)	(SDiazMesSe_135F3I3B)	(SDiazMesS_IF5B)	(2SDiazMesSe_5F5IB)
Crystal data				
Chemical formula	$3(C_6F_3I_3)\cdot3(C_{23}H_{30}N_2S)$	$C_6F_3I_3\cdot C_{23}H_{30}N_2Se$	$C_6F_5I\cdot C_{23}H_{30}N_2S$	$5(C_6F_5I)\cdot2(C_{23}H_{30}N_2Se)$
M_r	2628.92	923.21	660.51	2296.70
Crystal system, space group	Monoclinic, $P2_1/c$	Monoclinic, $P2_1/n$	Orthorhombic, $Pna2_1$	Monoclinic, $P2_1/c$
Temperature (K)	100	100	100	100
a, *b*, *c* (Å)	39.226 (4), 7.9811 (9), 30.341 (4)	8.0412 (6), 30.070 (2), 12.4774 (10)	7.8472 (5), 16.1751 (13), 21.7956 (19)	25.9292 (14), 8.0709 (4), 20.4309 (11)
α, β, γ (°)	90, 106.162 (4), 90	90, 92.632 (3), 90	90, 90, 90	90, 113.037 (2), 90
V (Å^3)	9123.3 (18)	3013.8 (4)	2766.5 (4)	3934.6 (4)
Z	4	4	4	2

Radiation type	Mo *K*α	Mo *K*α	Mo *K*α	Mo *K*α
μ (mm^{-1})	3.19	4.36	1.29	3.01
Crystal size (mm)	0.34 × 0.05 × 0.05	0.19 × 0.06 × 0.05	0.41 × 0.06 × 0.03	0.24 × 0.19 × 0.13
Data collection				
Diffractometer	Bruker D8 Venture Photon 2	Bruker D8 Venture Photon 2	Bruker D8 Venture Photon 2	Bruker D8 Venture Photon 2
Absorption correction	Multi-scan *SADABS* v2016/2 (Bruker AXS Inc., 2017)	Multi-scan *SADABS* v2016/2 (Bruker AXS Inc., 2017)	Multi-scan *SADABS* v2016/2 (Bruker AXS Inc., 2017)	Multi-scan *SADABS* v2016/2 (Bruker AXS Inc., 2017)
T_{min}, T_{max}	0.625, 0.745	0.617, 0.746	0.632, 0.746	0.662, 0.746
No. of measured, independent and observed [$I > 2\sigma(I)$] reflections	105324, 17944, 15994	42494, 6919, 5611	30210, 6227, 5489	61094, 9058, 8222
R_{int}	0.054	0.058	0.048	0.035
$(\sin \theta/\lambda)_{max}$ (Å^{-1})	0.618	0.650	0.652	0.650
Refinement				
$R[F^2 > 2\sigma(F^2)]$, $wR(F^2)$, S	0.053, 0.117, 1.27	0.031, 0.064, 1.17	0.028, 0.063, 1.12	0.024, 0.053, 1.09
No. of reflections	17944	6919	6227	9058

No. of parameters	1064	349	349	565
No. of restraints	36	0	1	51
H-atom treatment	H-atom parameters constrained	H-atom parameters constrained	H-atom parameters constrained	H-atom parameters constrained
	$w = 1/[\sigma^2(F_o^2) + 146.2483P]$ where $P = (F_o^2 + 2F_c^2)/3$	$w = 1/[\sigma^2(F_o^2) + (0.0047P)^2 + 7.9038P]$ where $P = (F_o^2 + 2F_c^2)/3$	$w = 1/[\sigma^2(F_o^2) + (0.0101P)^2 + 2.3147P]$ where $P = (F_o^2 + 2F_c^2)/3$	$w = 1/[\sigma^2(F_o^2) + (0.0103P)^2 + 7.6201P]$ where $P = (F_o^2 + 2F_c^2)/3$
$\Delta\rho_{max}$, $\Delta\rho_{min}$ (e Å^{-3})	1.36, −1.13	0.78, −0.75	0.68, −0.52	1.06, −0.75
Absolute structure	-	-	Flack x determined using 2370 quotients [(I+)-(I-)]/[(I+)+(I-)] (Parsons, Flack and Wagner, Acta Cryst. B69 (2013) 249-259).	-
Absolute structure parameter	-	-	−0.027 (12)	-

	(2SDiazMesS_TIE)	(2SDiazMesSe_TIE)
Crystal data		
Chemical formula	$CI_2·C_{23}H_{30}N_2S$	$C_2I_4·2(C_{23}H_{30}N_2Se)$
M_r	632.36	1358.52

Crystal system, space group	Triclinic, *P*-1	Triclinic, *P*-1
Temperature (K)	100	100
a, *b*, *c* (Å)	7.9170 (6), 11.6457 (8), 15.0306 (10)	7.9777 (5), 11.7334 (7), 15.0059 (9)
α, β, γ (°)	67.358 (2), 83.953 (2), 72.067 (2)	67.540 (2), 82.671 (2), 71.632 (2)
V (Å^3)	1216.73 (15)	1231.91 (13)
Z	2	1
Radiation type	Mo *K*α	Mo *K*α
μ (mm^{-1})	2.68	4.04
Crystal size (mm)	0.23 × 0.13 × 0.05	0.14 × 0.09 × 0.08
Data collection		
Diffractometer	Bruker D8 Venture Photon 2	Bruker D8 Venture Photon 2
Absorption correction	Multi-scan *SADABS* v2016/2 (Bruker AXS Inc., 2017)	Multi-scan *SADABS* v2016/2 (Bruker AXS Inc., 2017)
T_{min}, T_{max}	0.598, 0.746	0.636, 0.746
No. of measured, independent and observed [$I > 2\sigma(I)$] reflections	38610, 6789, 6164	41652, 6224, 5668
R_{int}	0.038	0.037
$(\sin \theta/\lambda)_{max}$ (Å^{-1})	0.696	0.671
Refinement		
$R[F^2 > 2\sigma(F^2)]$, $wR(F^2)$, S	0.032, 0.070, 1.06	0.030, 0.065, 1.08
No. of reflections	6789	6224
No. of parameters	384	384

No. of restraints	30	36
H-atom treatment	H-atom parameters constrained	H-atom parameters constrained
	$w = 1/[\sigma^2(F_o^2) + 5.6607P]$ where $P = (F_o^2 + 2F_c^2)/3$	$w = 1/[\sigma^2(F_o^2) + 5.9779P]$ where $P = (F_o^2 + 2F_c^2)/3$
$\Delta\rho_{max}$, $\Delta\rho_{min}$ (e Å^{-3})	3.79, −3.62	3.78, −3.88
Absolute structure	-	-
Absolute structure parameter	-	-

Computer programs: *APEX3* v2017.3-0 (Bruker AXS Inc., 2017), *SAINT* V8.38A (Bruker AXS Inc., 2017), SHELXT 2018/2 (Sheldrick, 2015*a*), *SHELXL* 2018/3 (Sheldrick, 2015*b*), *Mercury* (Macrae *et al.*, 2008), Olex2 1.5 (Dolomanov *et al.*, 2009).

Table S3 Hydrogen-bond geometry (Å, º) for (SDiazMesS)

D—H···*A*	*D*—H	H···*A*	*D*···*A*	*D*—H···*A*
C3—H3*B*···S1^{i}	0.99	3.01	3.7030 (15)	128
C5—H5*A*···S1^{i}	0.99	2.87	3.4858 (14)	121

Symmetry code: (i) *x*, −*y*+3/2, *z*+1/2.

Table S4 Hydrogen-bond geometry (Å, º) for (SDiazMesSe)

D—H···*A*	*D*—H	H···*A*	*D*···*A*	*D*—H···*A*
C2—H2*B*···Se1^{i}	0.99	3.02	3.671 (2)	125
C3—H3*A*···Se1^{i}	0.99	2.88	3.665 (2)	137
C4—H4*B*···Se2^{ii}	0.99	3.12	3.641 (2)	114
C5—H5*A*···Se2^{ii}	0.99	3.09	3.754 (2)	126
C26—H26*B*···Se2^{iii}	0.99	2.94	3.796 (2)	146

Symmetry codes: (i) −*x*+1/2, *y*+1/2, *z*; (ii) −*x*+1, −*y*+1, −*z*+1; (iii) −*x*+1/2, *y*−1/2, *z*.

Table S5 Hydrogen-bond geometry (Å, º) for (SDiazMesS_MeCN)

D—H···*A*	*D*—H	H···*A*	*D*···*A*	*D*—H···*A*
C4—H4*B*···S1^{i}	0.99	2.92	3.6972 (17)	136
C25—H25*B*···S1^{ii}	0.98	2.92	3.8180 (17)	152

Symmetry codes: (i) −*x*+1, *y*−1/2, −*z*+3/2; (ii) −*x*+1, −*y*+1, −*z*+1.

Table S6 Hydrogen-bond geometry (Å, º) for (SDiazMesSe_MeCN)

D—H···*A*	*D*—H	H···*A*	*D*···*A*	*D*—H···*A*
C4—H4*A*···Se1^{i}	0.99	3.09	4.009 (6)	155
C4—H4*B*···Se1^{ii}	0.99	2.91	3.737 (4)	141
C4*B*—H4*BB*···Se1^{ii}	0.99	3.15	3.965 (3)	141
C25—H25*B*···Se1^{iii}	0.98	3.03	3.829 (2)	140

Symmetry codes: (i) *x*, −*y*+1/2, *z*+1/2; (ii) −*x*+1, *y*−1/2, −*z*+3/2; (iii) −*x*+1, −*y*+1, −*z*+1.

Table S7 Hydrogen-bond geometry (Å, º) for (SDiazMesSI2)

D—H···*A*	*D*—H	H···*A*	*D*···*A*	*D*—H···*A*
C2—H2*B*···I1^{i}	0.99	3.16	4.111 (4)	162
C4—H4*B*···I2^{ii}	0.99	3.16	4.034 (4)	148
C5—H5*A*···I1^{i}	0.99	3.10	3.896 (3)	138

Symmetry codes: (i) *x*+1, *y*, *z*; (ii) *x*+1, *y*−1, *z*.

Table S8 Hydrogen-bond geometry (Å, º) for (SDiazMesSeI2)

D—H···*A*	*D*—H	H···*A*	*D*···*A*	*D*—H···*A*
C2—H2*A*···I1^{i}	0.99	3.08	3.885 (2)	139
C3—H3*B*···I2^{ii}	0.99	3.14	4.007 (2)	147
C5—H5*B*···I1^{i}	0.99	3.11	4.067 (2)	164

Symmetry codes: (i) *x*+1, *y*, *z*; (ii) *x*+1, *y*−1, *z*.

Table S9 Hydrogen-bond geometry (Å, º) for (SDiazMesSI2_I2)

D—H···*A*	*D*—H	H···*A*	*D*···*A*	*D*—H···*A*
C2—H2*B*···I4^{i}	0.99	3.22	3.7794 (18)	118
C5—H5*A*···I2^{ii}	0.99	3.26	4.0837 (19)	142

Symmetry codes: (i) *x*+1, *y*, *z*; (ii) *x*+1, *y*−1, *z*.

Table S10 Hydrogen-bond geometry (Å, º) for (SDiazMesSeI_I3)

D—H···*A*	*D*—H	H···*A*	*D*···*A*	*D*—H···*A*
C2—H2*A*···I3^{i}	0.99	3.24	4.029 (10)	138
C2—H2*B*···I4^{i}	0.99	3.12	3.823 (11)	129
C3—H3*B*···I2^{ii}	0.99	3.22	3.998 (11)	137
C5—H5*B*···Se1^{iii}	0.99	3.09	3.693 (11)	121

Symmetry codes: (i) −*x*+1, −*y*+1, −*z*+1; (ii) *x*−1, *y*, *z*; (iii) *x*−1/2, −*y*+3/2, *z*−1/2.

Table S11 Hydrogen-bond geometry (Å, º) for (SDiazMesSeI_I3_DCM)

D—H···*A*	*D*—H	H···*A*	*D*···*A*	*D*—H···*A*
C2—H2*B*···I3^{i}	0.99	3.24	3.755 (2)	114
C4—H4*A*···I3^{ii}	0.99	3.15	3.748 (2)	120
C5—H5*A*···I1^{i}	0.99	3.09	3.930 (2)	144

Symmetry codes: (i) *x*−1, *y*, *z*; (ii) *x*−1, *y*+1, *z*.

Table S12 Hydrogen-bond geometry (Å, º) for (SDiazMesSeDMK_I3_I2)

D—H···*A*	*D*—H	H···*A*	*D*···*A*	*D*—H···*A*
C2—H2*A*···I2^{i}	0.99	3.29	4.274 (10)	173
C3—H3*B*···I2^{ii}	0.99	3.06	3.674 (14)	122
C4—H4*B*···O1^{iii}	0.99	2.32	3.081 (13)	133
C26—H26*B*···I6^{iv}	0.98	3.26	4.186 (13)	158

Symmetry codes: (i) −*x*+1, −*y*+1, −*z*+1; (ii) *x*−1/2, *y*+1/2, *z*; (iii) *x*−1/2, *y*−1/2, *z*; (iv) *x*+1/2, *y*+1/2, *z*.

Table S13 Hydrogen-bond geometry (Å, º) for (SDiazMesSMIBK_I3)

D—H···*A*	*D*—H	H···*A*	*D*···*A*	*D*—H···*A*
C2—H2*A*···I1	0.99	3.07	3.693 (3)	122
C2—H2*B*···I2	0.99	3.17	3.986 (3)	141

Table S14 Hydrogen-bond geometry (Å, º) for (SDiazMesSeMIBK_I3)

D—H···*A*	*D*—H	H···*A*	*D*···*A*	*D*—H···*A*
C2—H2*A*···I3	0.99	3.10	3.690 (2)	120
C2—H2*B*···I2	0.99	3.16	4.003 (2)	144
C3—H3*A*···O1^{i}	0.99	2.63	3.560 (3)	156

Symmetry code: (i) *x*+1, *y*, *z*.

Table S15 Hydrogen-bond geometry (Å, º) for (SDiazMesS_12F4DIB)

D—H···*A*	*D*—H	H···*A*	*D*···*A*	*D*—H···*A*
C2—H2*B*···I2^{i}	0.99	3.11	4.041 (6)	156
C3—H3*A*···S1^{ii}	0.99	2.94	3.553 (7)	121

Symmetry codes: (i) −*x*+1, −*y*+1, *z*+1/2; (ii) *x*−1, *y*, *z*.

Table S16 Hydrogen-bond geometry (Å, º) for (SDiazMesS_13F4DIB)

D—H···*A*	*D*—H	H···*A*	*D*···*A*	*D*—H···*A*
C2—H2*A*···I2^{i}	0.99	3.19	3.897 (12)	129
C5—H5*A*···F4	0.99	2.62	3.330 (11)	129
C2*B*—H2*BB*···I1^{ii}	0.99	3.26	4.115 (12)	146
C4*B*—H4*BA*···F3^{iii}	0.99	2.48	3.247 (13)	134
C5*B*—H5*BB*···F4	0.99	2.62	3.153 (12)	114

Symmetry codes: (i) −*x*+3/2, −*y*+1, *z*+1/2; (ii) *x*+1/2, −*y*+3/2, −*z*+1; (iii) *x*+1/2, −*y*+1/2, −*z*+1.

Table S17 Hydrogen-bond geometry (Å, º) for (SDiazMesSe_13F4DIB)

D—H···*A*	*D*—H	H···*A*	*D*···*A*	*D*—H···*A*

C2—H2*B*···I2[i]	0.99	3.22	3.947 (6)	132
C2*B*—H2*BA*···I1[ii]	0.99	3.28	4.124 (12)	145
C3*B*—H3*BA*···Se1[iii]	0.99	3.03	3.701 (16)	127
C4*B*—H4*BB*···F3[iv]	0.99	2.42	3.214 (13)	137
C5—H5*B*···F4	0.99	2.55	3.299 (6)	132
C5*B*—H5*BA*···F4	0.99	2.59	3.119 (11)	114

Symmetry codes: (i) −*x*+1/2, −*y*+1, *z*−1/2; (ii) *x*−1/2, −*y*+1/2, −*z*+1; (iii) *x*−1, *y*, *z*; (iv) *x*−1/2, −*y*+3/2, −*z*+1.

Table S18 Hydrogen-bond geometry (Å, º) for (2SDiazMesS_13F4DIB)

D—H···*A*	*D*—H	H···*A*	*D*···*A*	*D*—H···*A*
C5—H5*B*···I1[i]	0.99	3.29	4.117 (5)	143
C3*B*—H3*BB*···S1[i]	0.99	2.85	3.574 (9)	131

Symmetry code: (i) *x*, *y*, *z*+1.

Table S19 Hydrogen-bond geometry (Å, º) for (2SDiazMesSe_13F4DIB)

D—H···*A*	*D*—H	H···*A*	*D*···*A*	*D*—H···*A*
C2—H2*B*···Se1[i]	0.99	2.93	3.850 (4)	155
C5—H5*A*···I1[ii]	0.99	3.30	4.150 (4)	145

Symmetry codes: (i) *x*, *y*, *z*+1; (ii) −*x*+1, −*y*+1, −*z*+1.

Table S20 Hydrogen-bond geometry (Å, º) for (2SDiazMesS_14F4DIB_t)

D—H···*A*	*D*—H	H···*A*	*D*···*A*	*D*—H···*A*
C2—H2*B*···S1[i]	0.99	2.88	3.609 (3)	131

Symmetry code: (i) *x*+1, *y*, *z*.

Table S21 Hydrogen-bond geometry (Å, º) for (2SDiazMesSe_14F4DIB_t)

D—H···*A*	*D*—H	H···*A*	*D*···*A*	*D*—H···*A*

C2—H2*B*···Se1[i]	0.99	2.83	3.5653 (13)	132

Symmetry code: (i) *x*+1, *y*, *z*.

Table S22 Hydrogen-bond geometry (Å, º) for (2SDiazMesS_14F4DIB_m)

D—H···*A*	*D*—H	H···*A*	*D*···*A*	*D*—H···*A*
C2—H2*A*···F2[i]	0.99	2.44	3.310 (2)	146
C2—H2*B*···S1[ii]	0.99	2.91	3.898 (2)	173

Symmetry codes: (i) *x*−1, *y*, *z*; (ii) *x*, −*y*+3/2, *z*+1/2.

Table S23 Hydrogen-bond geometry (Å, º) for (SDiazMesS_135F3I3B)

D—H···*A*	*D*—H	H···*A*	*D*···*A*	*D*—H···*A*
C2—H2*A*···I6[i]	0.99	3.30	4.234 (9)	158
C4—H4*A*···S1[ii]	0.99	2.88	3.548 (10)	125
C5—H5*B*···F6[i]	0.99	2.58	3.365 (11)	136
C25—H25*B*···I5[iii]	0.99	3.12	3.960 (13)	144
C26—H26*B*···I7[iii]	0.99	3.13	3.854 (11)	131
C27—H27*B*···F3[iv]	0.99	2.48	3.283 (11)	138
C25*B*—H25*C*···I7[iii]	0.99	3.24	4.06 (3)	141
C26*B*—H26*D*···S2[iii]	0.99	2.85	3.60 (3)	134
C50—H50*A*···I9[v]	0.99	3.07	3.843 (9)	136
C51—H51*B*···I3	0.99	3.13	4.083 (9)	162

Symmetry codes: (i) −*x*, *y*+1/2, −*z*+1/2; (ii) *x*, *y*+1, *z*; (iii) *x*, *y*−1, *z*; (iv) *x*, −*y*+1/2, *z*+1/2; (v) −*x*+1, −*y*+1, −*z*+1.

Table S24 Hydrogen-bond geometry (Å, º) for (SDiazMesSe_135F3I3B)

D—H···*A*	*D*—H	H···*A*	*D*···*A*	*D*—H···*A*
C4—H4*A*···I2[i]	0.99	3.17	3.899 (4)	132
C4—H4*A*···Se1[ii]	0.99	3.04	3.771 (4)	132

C5—H5*B*···I1	0.99	3.08	4.051 (4)	167

Symmetry codes: (i) *x*, *y*, *z*+1; (ii) *x*−1, *y*, *z*.

Table S25 Hydrogen-bond geometry (Å, º) for (2SDiazMesSe_5F5IB)

D—H···*A*	*D*—H	H···*A*	*D*···*A*	*D*—H···*A*
C2—H2*A*···I2^{i}	0.99	3.25	3.852 (3)	121
C3—H3*B*···F12^{i}	0.99	2.54	3.456 (4)	155
C5—H5*B*···F4^{ii}	0.99	2.55	3.530 (3)	170

Symmetry codes: (i) *x*, −*y*+1/2, *z*−1/2; (ii) −*x*+1, *y*+1/2, −*z*+1/2.

Table S26 Hydrogen-bond geometry (Å, º) for (2SDiazMesS_TIE)

D—H···*A*	*D*—H	H···*A*	*D*···*A*	*D*—H···*A*
C4*B*—H4*BB*···S1^{i}	0.99	2.97	3.72 (3)	133

Symmetry code: (i) *x*−1, *y*, *z*.

Table S27 Hydrogen-bond geometry (Å, º) for (2SDiazMesSe_TIE)

D—H···*A*	*D*—H	H···*A*	*D*···*A*	*D*—H···*A*
C4*A*—H4*AB*···Se1^{i}	0.99	2.97	3.73 (3)	134

Symmetry code: (i) *x*−1, *y*, *z*.
